# Hyaluronan synthase 3 deficiency lowers the incidence of ruptures of abdominal aortic aneurysms by reducing monocyte infiltration

**DOI:** 10.3389/fimmu.2025.1680246

**Published:** 2025-11-06

**Authors:** Viola Niemann, Fedor Brack, Luca Rolauer, Janet Kaczur, Patrick Petzsch, Karl Köhrer, Christine Quast, Norbert Gerdes, Pascal Bouvain, Katharina Voigt, Martina Krüger, Alexander Brückner, Bernd K. Fleischmann, Daniela Wenzel, Philipp Barnowski, Laura-Marie Zimmermann, Sakine Simsekyilmaz, Timm Filler, Wiebke Ibing, Tobias Feige, Kim J. Krott, Markus U. Wagenhäuser, Jens W. Fischer, Margitta Elvers, Gerhard Sengle, Ulrich Flögel, Christian Hundhausen, Tatsiana Suvorava, Maria Grandoch

**Affiliations:** 1 Institute of Translational Pharmacology, Medical Faculty and University Hospital Düsseldorf, Heinrich Heine University Düsseldorf, Düsseldorf, Germany; 2 Genomics and Transcriptomics Laboratory (GTL), Biological and Medical Research Center (BMFZ), Medical Faculty and University Hospital Düsseldorf, Heinrich Heine University Düsseldorf, Düsseldorf, Germany; 3 Division of Cardiology, Pulmonology, and Vascular Medicine, Medical Faculty and University Hospital Düsseldorf, Heinrich Heine University Düsseldorf, Düsseldorf, Germany; 4 CARID, Cardiovascular Research Institute Düsseldorf, Medical Faculty and University Hospital Düsseldorf, Heinrich Heine University Düsseldorf, Düsseldorf, Germany; 5 Institute of Molecular Cardiology, Medical Faculty and University Hospital Düsseldorf, Heinrich Heine University Düsseldorf, Düsseldorf, Germany; 6 Institute for Cardiovascular Physiology, Medical Faculty and University Hospital Düsseldorf, Heinrich Heine University, Düsseldorf, Germany; 7 Institute of Physiology I, Life and Brain Center, Medical Faculty and University of Bonn, Bonn, Germany; 8 Department of Systems Physiology, Medical Faculty, Ruhr University of Bochum, Bochum, Germany; 9 Department of Pediatrics and Adolescent Medicine, Medical Faculty and University Hospital Cologne, University of Cologne, Cologne, Germany; 10 Center for Molecular Medicine Cologne (CMMC), University of Cologne, Cologne, Germany; 11 Center for Biochemistry, Medical Faculty and University Hospital Cologne, University of Cologne, Cologne, Germany; 12 Cologne Center for Musculoskeletal Biomechanics (CCMB), Medical Faculty and University Hospital Cologne, University of Cologne, Cologne, Germany; 13 Cologne Excellence Cluster on Cellular Stress Responses in Ageing-Associated Diseases (CECAD), University of Cologne, Cologne, Germany; 14 Institute of Pharmacology, Medical Faculty and University Hospital Düsseldorf, Heinrich-Heine-University Düsseldorf, Düsseldorf, Germany; 15 Department of Clinical Anatomy I, Medical Faculty and University Hospital Düsseldorf, Heinrich Heine University Düsseldorf, Düsseldorf, Germany; 16 Department of Vascular- and Endovascular Surgery, University Hospital Düsseldorf, Heinrich Heine University, Düsseldorf, Germany; 17 Freelance Consultant, Düsseldorf, Germany

**Keywords:** aortic dissection, hyaluronan synthase 3, inflammation, myeloid leukocytes, recruitment

## Abstract

**Introduction:**

Abdominal aortic aneurysms and dissections (AAA/AD) are vascular disorders with high mortality due to aortic ruptures. Critical pathomechanisms involve immune cell infiltration and degradation of the vascular extracellular matrix (ECM). Hyaluronan (HA), a major constituent of the ECM synthesized by three HA synthase isoenzymes (HAS1-3), plays a role in both processes. Specifically, HAS3 is crucially involved in inflammatory conditions. Here, we aimed to elucidate the role of HAS3-derived HA in AAA/AD.

**Methods:**

Mice double-deficient for *apolipoprotein E* and *Has3* (*Apoe/Has3*-DKO) and littermate controls (*Apoe*-KO) were studied in a model of angiotensin II (AngII)-induced AAA/AD.

**Results:**

*Has3* deficiency improved survival in *Apoe/Has3*-DKO mice *via* reducing aortic ruptures. This was associated with decreased monocyte infiltration into the vessel wall. Aortic RNA-Seq analysis indicated disturbed immune cell adhesion and diapedesis. Transfer of *Apoe-*deficient bone marrow into *Apoe/Has3*-DKO mice largely normalized the *Apoe/Has3*-DKO phenotype. While gene expression in endothelial cells (ECs) was not affected, AngII-induced upregulation of proinflammatory cytokines, adhesion receptors and the HA receptor CD44 was attenuated in *Apoe/Has3-*DKO monocytes. This reduced CD44 cell surface expression in *Apoe/Has3-*double-deficient monocytes, ultimately inhibiting their *in vitro* transmigration.

**Discussion:**

Our results show that HAS3 plays a key role in AAA/AD formation and suggest the HAS3/CD44 axis as promising therapeutic target to reduce monocyte recruitment and aortic rupture.

## Introduction

Aortic aneurysms are a common vascular disease associated with a high risk of morbidity and mortality (1, 2). The pathophysiology of abdominal aortic aneurysm (AAA) formation, a dilatation mostly occurring between the renal arteries and the femoral bifurcation, includes diverse genetic and environmental risk factors such as age, gender and smoking. As disease progresses, the aortic wall thins and becomes susceptible to dissection. Intimal tears can extend into the media, forming a false lumen that may or may not communicate with the true lumen (3). Progressive delamination of the wall can ultimately result in rupture, causing fatal bleeding. AAA formation is characterized by continuous extracellular matrix (ECM) degradation, smooth muscle cell (SMC) apoptosis and phenotypic switching (4, 5), as well as immune cell infiltration leading to elastic fiber fragmentation (6). The vascular hyaluronan (HA)-rich matrix is central to these processes, regulating vascular function and maintaining aortic homeostasis (7). HA, a glycosaminoglycan synthesized by three HA synthase isoenzymes (HAS1-3), is present in all layers of the vascular wall - most abundantly in the intima and adventitia - and influences phenotypic modulation of multiple cell types. Among the isoenzymes, HAS3 has been extensively studied in cardiovascular pathologies such as atherogenesis (8) and neointima hyperplasia (9), where it promotes proinflammatory responses (8–12). Inflammation is a critical driver of AAA pathogenesis, mediated by leukocyte recruitment to the vascular wall. During the initial phase of AAA development, monocytes from the spleen and bone marrow infiltrate the aortic wall *via* endothelial binding, guided by chemokines and receptors such as CCR2, CXCR1, and selectins (13). Recently, triggering receptor expressed on myeloid cells (TREM-1) was identified as a key mediator promoting mobilization of classical Ly6Chigh monocytes from the spleen, highlighting subset-specific responses (14). Clinical studies also report substantial changes in peripheral monocyte subsets and phenotypes during AAA formation (15), as well as in abdominal AD patients (16), suggesting that distinct monocyte populations contribute to vascular inflammation, remodeling, and disease progression. In inflammatory conditions, HA is crucial for shaping the immune response, as the HA-rich matrix is not only a passive component of the microenvironment but also actively regulates local immune functions. Thus, under a variety of inflammatory disease states, a pro-inflammatory HA-rich ECM is synthesized providing an adhesive matrix for monocytes and T cells (3, 17). In addition, HA fragments can directly activate monocyte/macrophages (18) and increase the expression of inflammation-related cytokines (19). Here, the interaction between HA and its receptors, such as CD44 and receptor of HA-mediated motility (RHAMM), can mediate the adhesion and migration of immune cells (18), adhesion of platelets (20) as well as the proliferation of SMCs (7, 9). Further, CD44 is important for proinflammatory gene expression that is regulated *via* HA-CD44 interactions (18). Indeed, an important pathophysiologic role for CD44 in the development of thoracic AA and AD has been described, which was associated with a reduction in neutrophil infiltration in the adventitia (21). Also, in human abdominal aneurysm increased CD44 expression has been reported for different cell types such as SMCs, endothelial cells and macrophages.

Since inflammation and immune cell invasion play such an important role in the development and progression of AAA, the HA-rich matrix is of crucial importance and could pave the way for new therapeutic approaches. The aim of this study was therefore to investigate the role of HA and in particular of HAS3, which is known to be involved in inflammatory processes, in the development of AAA/AD.

## Methods

### Mice

All experimental procedures and animal care were approved by the local animal experimentation ethics committee (LANUV, State Agency for Nature, Environment and Consumer Protection, file number 81-02.04.2018.A222 and 81-02.04.2023.A187), and performed in accordance with the guidelines of German Animal Welfare Law and guidelines from Directive 2010/63/EU of the European Parliament on the protection of animals used for scientific purposes. Male mice double-deficient for apolipoprotein E (*Apoe*) and hyaluronan synthase 3 (*Has3*) (*Apoe/Has3*-DKO) and respective control mice (*Apoe*-KO) were used in this study. For *Apoe/Has3*-DKO mice *Has3*-deficient mice generated by genOway (Lyon Cedex, France) as described by (9) were crossbred with *Apoe*-KO mice (Taconic, Hudson, NY, USA). In some experiments, single deficient *Has3*-KO mice (9) and their wild type controls (*Has3*-WT) were used. Transgenic mice of each genotype were randomly allocated to receive AngII infusion or placebo. Group identity was masked during outcome assessment and analysis; unblinding occurred after primary analyses. For the bone marrow transfer experiments, treatment administration could not be blinded due to handling requirements. Mice of both genotypes were stratified by body weight into two equal groups, serving either as controls or as recipients of treatment. Outcome assessment and data analysis were conducted under blinded conditions, and unblinding occurred only after analysis.

*Apoe*-KO and *Apoe/Has3*-DKO mice were fed a western-type diet (WD) containing 21% fat and 0.15% cholesterol (S8200-E010, Ssniff Spezialdiäten GmbH, Soest, Germany) starting from the day of AngII infusion. *Has3*-KO and *Has3*-WT mice were kept on maintenance diet (V1534-300, Ssniff Spezialdiäten GmbH, Soest, Germany) and obtained β-Aminopropionitrile (BAPN, 0.1% in drinking water InvitroGen Thermo Fischer, Carlsbad, CA, USA) for 3 days prior to and throughout the duration of AngII infusion. Mice were inspected daily and excluded from the experiment when certain criteria of suffering were observed. At the end of the experiments, mice were euthanized by CO_2_ inhalation, blood was collected by cardiac puncture and organs were harvested for further analysis.

### AngII infusion model of AAA/AD

Eight to twelve-week-old *Apoe*-KO and *Apoe/Has3*-DKO mice were infused with AngII (Sigma-Aldrich, St. Louis, USA) or saline using osmotic minipumps (models 1003D, 1007D and 1004, Alzet, Cupertino, USA) at 1000 ng/kg/min as described by Trachet et al. (22) for 3, 7 and 28 days. In some experimental sets, eight to twelve-week-old *Has3*-KO and *Has3*-WT mice were infused with AngII for 7 days (1007D, Alzet, Cupertino, USA).

### Bone marrow transfer

Recipient *Apoe*-KO and *Apoe/Has3*-DKO were lethally irradiated with a dose of 10 Gy from a *^137^Cs* source that was delivered within 150s using the Biobeam GM 2000 (Gamma-Service Medical GmbH, Leipzig, Germany). Bone marrow cells were obtained from the tibia and femur of donor mice. *Apoe*-KO and *Apoe/Has3*-DKO mice were used as donors for each genotype to obtain the respective controls. Irradiated recipients were transplanted with 5.5×10^6^ bone-marrow cells per mouse *via* retro-orbital injection. Ten weeks after irradiation, osmotic mini pumps containing AngII (1000 ng/kg/min) were implanted, and mice were placed on WD for 3 or 28 days until organ harvest. Because leukocyte yields from the aorta were low, the entire aorta was allocated to enzymatic digestion for flow cytometry, precluding concurrent histology in this cohort.

### Blood pressure measurement by telemetry

Under ketamine/xylazine anesthesia mice were surgically implanted with a microminiaturized electronic pressure-sensing catheters (PA-C10, Data Sciences International (DSI), St. Paul, MN, USA) into the left common carotid artery. Mice were allowed a 7-day post-surgery stabilization period before starting the acquisition of hemodynamic data. After 3 days of baseline measurement, AngII pumps were implanted, and blood pressure and heart rate measurements were collected continuously with sampling every 20 min for 10 s intervals.

### Quantification of aneurysm development and progression *in vivo* by ultrasonography

Mice were monitored for aortic aneurysm formation at baseline and three days, one week, two weeks, three weeks and four weeks after implantation of minipumps with AngII infusion using a Vevo 3100 high-resolution micro-imaging system and a 20–46 MHz - transducer (MX400, VisualSonics Inc., FUJIFILM, Toronto, Canada). In the abdominal aorta ultrasound images were captured in long axis view to visualize entire length of the abdominal aorta and perform initial estimation of size, shape and location of the aneurysm. Short axis view was used to measure maximal aortic diameter leading to leading edge and lumen area as maximum transverse dimension orthogonal to the vessel axis. All measurements were performed at mid-systole, when the aorta is maximally and visually dilated. Color Doppler was used to visualize the blood flow pattern and aortic lumen in AAA. Pulsed wave (PW) Doppler was used to measure the velocity of blood either in suprarenal aorta between celiac artery and superior mesenteric artery or the largest portion of aneurysm. Vevo3100 software (VevoLAB, FUJIFILM, Visual Sonics, Toronto, Canada) was used to calculate maximal systolic diameter, centerline peak blood flow velocity, visualize true and false lumen.

### Magnetic resonance imaging

Experiments were performed at a vertical 9.4 T Bruker AVANCE^III^ Wide Bore nuclear magnetic resonance (NMR) spectrometer (Bruker, Ettlingen, Germany) operating at frequencies of 400.21 MHz for ^1^H and 376.54 MHz for ^19^F measurements using microimaging units as described previously (23). Data were acquired using a 25-mm quadrature resonator tuneable to ^1^H and ^19^F. After acquisition of the morphological ^1^H images, the resonator was tuned to ^19^F and anatomically matching ^19^F images were recorded. The reference power and the receiver gain were kept constant between the measurements to ensure comparability of the ^19^F scans.

To conduct ^1^H/^19^F magnetic resonance imaging (MRI), perfluorocarbon nanoemulsions (PFCs) were prepared as described previously (23) and injected intravenously (3 mmol/kg/body weight (BW)) *via* the tail vein in anesthetized mice. To visualize the anatomy of the region of interest, ^1^H MR reference images from the abdomen were acquired using a rapid acquisition and relaxation enhancement sequence [RARE; field of view (FOV) = 2.56 × 2.56 cm^2^, matrix = 256 × 256, 0.1 × 0.1 mm^2^ in plane resolution, 1 mm slice thickness (ST); repetition time (TR) = 2,500 ms; RARE factor = 16, 6 averages (NA), acquisition time (TAcq) = ~5 min] as described previously (23). Anatomically matching ^19^F images were recorded from the same FOV with a ^19^F RARE sequence (matrix = 64 × 64, 0.4 × 0.4 mm^2^ in plane resolution, ST = 1 mm, TR = 2,500 ms, RARE factor = 32, NA = 256, and TAcq = 21 min).

### Transthoracic echocardiography

To determine cardiac function parameters, *Apoe*-KO and *Apoe/Has3*-DKO mice were examined echocardiographically before AngII infusion and 3 and 7 days after AngII infusion using the Vevo 3100 high-resolution micro-imaging system with a 20–46 MHz transducer (MX400, VisualSonics Inc., FUJIFILM, Toronto, Canada). During the entire measurement, the heart rate, respiratory rate and ECG were recorded *via* a heated electrode contact pad. In addition, the body temperature was monitored using a rectal temperature measurement and maintained at 37 °C. Parasternal long-axis view was acquired. Left ventricular (LV) end-systolic and end-diastolic volumes (ESV and EDV) were calculated by identification of frames with maximal and minimal cross-sectional areas. LV ejection fraction (LVEF), cardiac output (CO) and stroke volume (SV) were calculated from the volume data.

### Preparation and passive stretching of aortic strips

Aorta was freshly isolated from *Apoe*-KO and *Apoe/Has3*-DKO-deficient mice after 7 days of AngII-infusion and stored at –20 °C in a solution containing 50% glycerol and 50% low ionic strength buffer (consisting of 75 mM potassium chloride, 10 mM Tris-HCl, 2 mM Mg-chloride, 2 mM EGTA, protease and phosphatase inhibitor cocktail a, pH 7.1) until use. For tension measurements, the aortic preparations were rinsed in relaxing solution (7.8 mM ATP, 20 mM creatine phosphate, 20 mM imidazole, 4 mM EGTA, 12 mM Mg-propionate, 97.6 mM K-propionate, pH 7.0, 30 mM 2,3-butanedione monoxime (BDM), 1 mM dithiothreitol (DTT), protease and phosphatase inhibitor cocktail ) and rings (approximately 800 µm thick), were then cut from the abdominal section between the diaphragm and the common iliac arteries. For tension measurements, the rings were opened to acquire a longitudinal strip preparation (approximately 1000 µm length). Force measurements were performed with a muscle mechanics workstation (Myotronic, Heidelberg, Germany) at room temperature. Aortic strips were bathed in relaxing solution (see above) and mounted to the motor arm and force transducer using stainless steel clips. Each tissue strip underwent 5 cycles with each 100 s stepwise stretching to 200% of its initial length, with a recovery phase of 60 s between measurements. Passive tension was recorded at the end of each stretch period and related to cross-sectional area.

### Measurement of endothelial function and vascular reactivity *ex vivo*

Aortas of *Apoe*-KO and *Apoe*/*Has3*-DKO were studied at baseline and on day 3 post AngII infusion. Aortas were cannulated and pressurized. Briefly, after an equilibration phase of 60 min, aortic rings were constricted twice by the administration of 80 mM KCl. The second vasoconstriction was taken as the maximal receptor-independent vasoconstriction. Function of the endothelium was examined by cumulative addition of acetylcholine (0.01–30 μM ACh) after sub-maximal precontraction with phenylephrine. After washing, vascular contractility to increasing concentrations of the α1-adrenergic receptor agonist phenylephrine (PE; 0.1 nM-10 µM) was determined. Subsequently, NO-dependent vasorelaxation to the NO donor diethylamine/nitric oxide (DEA/NO, 1–10 μM) was measured. Relaxation is expressed as percentage of phenylephrine-induced tone, vasoconstriction is shown as percentage of maximal KCl-induced contraction.

### Tropoelastin staining

Tropoelastin staining was performed on paraffin-embedded aorta. For the dewaxing of tissue sections, the slides were rehydrated with a decreasing alcohol concentration series. Paraformaldehyde (PFA, Carl Roth, Karlsruhe, Germany)-fixed tissue preparations were subjected to epitope retrieval After a tris-buffered saline (TBS) washing step and a one-hour blocking step with 5 % bovine serum albumin (BSA, Sigma-Aldrich, St. Louis, USA)/0.1 % Triton X-100 (Sigma-Aldrich, St. Louis, USA) in 1 × TBS, the sections were incubated at 4 °C overnight with the primary rabbit polyclonal anti-tropoelastin antibody solution (ab21600, Abcam, Cambridge, UK) 1:200 in 0.5 % BSA in TBS. After two TBS-Tween 0.1 % (Sigma-Aldrich) washing steps and a TBS washing step, the secondary anti-rabbit antibody (InvitroGen Thermo Fischer, Carlsbad, CA, USA), 1:1000 in 0.5 % BSA in TBS was applied for 1 h at room temperature.

### Second harmonic generation microscopy

For collagen detection, 5 µm cross sections of paraffin embedded aortas were dewaxed, and SHG images of aortic collagens were obtained by using a Leica TCS SP8 microscope (Leica microsystems, Wetzlar, Germany) with a Chameleon Vision II laser. SHG and two-photon-excited fluorescence (TPEF) were excited at 810 nm for detection at 440 nm and imaged with an IR Apo L25x/0.95 W objective lens.

### Immunofluorescence staining of platelets in murine aortic tissue

Paraffin-embedded aortic tissue was sliced into 5 µm sections using an automatic microtome (Microm HM355, Thermo Fisher Scientific). Prior to staining, all sections were deparaffinised and hydrated. For antigen unmasking, the tissue sections were heated at 300 W in citrate buffer (pH 6.0) for 10 min. For staining of platelets, the aortic tissue sections were blocked for 1 h at room temperature with a protein blocking solution (#X0909, Dako). After blocking, the sections were specifically stained for platelets (GPIbα (CD42b), #M042-0, Emfret Analytics, 1:50) at 4 °C overnight. The respective IgG primary antibody served as negative control (#C301, Emfret Analytics, 1:50). The sections were incubated with a biotinylated secondary antibody (#BA-9400, Vector, 1:200) for 1 h at room temperature and afterwards with a streptavidin eFluorTM 660 conjugate (#50-4317-80, Thermo Fisher Scientific, 1:20) for 30 min at room temperature. For visualising nuclei, all tissue sections were stained with DAPI (4′, 6-Diamidine-2′-phenylindole dihydrochloride, #10236276001, Roche, 1:3000). Image generation was conducted using an Axio Observer.D1 microscope (Zeiss). Tissue sections of AngII infused mice were analysed as percentage of cell content normalised to the total aortic tissue, using Image J (version 1.53t) by applying the preinstalled “*Default*” threshold.

### Isolation of bone marrow-derived monocytes

Isolation of primary bone marrow monocytes from femurs and tibias of 10–12-week-old *Apoe*-KO and *Apoe/Has3*-DKO mice was performed using the CD115 MicroBead Kit, mouse (Miltenyi Biotec, Bergisch Gladbach, Germany) according to the manufacturer’s instructions (24). Monocytes were cultured in 5.5 mmol/L glucose Dulbecco's Modified Eagle Medium (DMEM) supplemented with 10 % fetal bovine serum (FBS), 15 mmol/L HEPES (Walkersville, USA), 100 U/ml penicillin and 100 μg/ml streptomycin (all reagents from Gibco Life Technologies, Paisley, UK) and incubated with AngII (10 ng/ml) or respective vehicle control for 6h. Subsequently, total RNA was isolated and gene expression analysis by quantitative real-time PCR (qPCR) was performed as described below.

### Monocyte transmigration assay

A transmigration assay was performed using mouse aortic endothelial cells (MAEC; Cat.-No. C57–6052 from Cell Biologics, Chicago, USA) seeded on Falcon^®^ Cell Culture inserts with 8μm pore size. Isolated bone marrow monocytes were isolated from femurs and tibias of *Apoe*-KO and *Apoe/Has3*-DKO mice on day 3 post AngII infusion as described above. Primary cells were resuspended in Roswell Park Memorial Institute (RPMI) 1640 medium supplemented with 10 % (v/v) FBS, 100 U/ml penicillin and 100 μg/ml streptomycin (all reagents from Gibco Life Technologies, Paisley, UK). Next, monocytes were incubated with calcein-AM (10µg/ml; Invitrogen, Thermo Fischer Scientific, Carlsbad, CA, USA) a membrane-permeable live-cell labeling dye for 30 min at room temperature and protected from the light.

Cells were washed with ice cold phosphate buffered saline (PBS), adjusted to 3-4 × 10^5^ cells/ml, resuspended in FBS and placed onto the filter. In a subset of experiments monocytes were first incubated with rat anti-mouse CD44 (10µg/ml; clone KM201, SouthernBiotech, Cat.-No. 1500-14) or respective IgG1 control isotype (KLH/G1-2-2, SouthernBiotech, Cat.-No. 0116-14) for 24 hours followed by seeding of the cells on inserts. The lower chamber was filled with medium supplemented with 50 ng/ml chemokine (C-C motif) ligand 2 (CCL2, R&D Systems, Minneapolis, Minnesota, USA). Medium without CCL2 was used as negative control. Monocytes were allowed to migrate for 4 hours at 37 °C. After this, the upper chamber was removed, and the calcein fluorescence in the lower chamber was counted by exciting at 490 nm and measuring fluorescence at 530 nm using a Synergy Microplate Reader (BioTek Instruments, Highland Park, USA). Transmigration of monocytes was measured in arbitrary units of mean fluorescence intensity.

### CD44 staining of human aorta

For quantification of CD44 in human aorta specimen, aortic tissue samples from patients with and without AAA were sliced into 5 µm sections using an automatic microtome. Prior to staining, all sections were deparaffinised and hydrated. For antigen unmasking, the tissue sections were heated at 300 W in citrate buffer (pH 6.0) for 30 min. Sections were specifically stained for CD44, (Sigma Aldrich, Cat.-No. HPA005785, 1:400) at 4 °C overnight. The sections were incubated with a donkey anti-rabbit IgG (H+L) cross-absorbed secondary antibody (Invitrogen, Cat.-No. 31458, 1:300) for 1 h at room temperature. After 10 min incubation in TBS buffer, antibody detection was performed with 3,3'-Diaminobenzidine (DAB, Zytomed Systems GmbH, DA-530). For visualising nuclei, all tissue sections were stained with hematoxylin. Images were acquired using a Leica DM 2000 LD super-resolution microscope. CD44 signals in tissue sections were analyzed as percentage of positive area fraction using ImageJ software (version 1.53t National Institute of Health, WI, USA).

### HA binding protein staining of human aorta

As described above, human aortic tissue samples were deparaffinised and hydrated. Control sections were digested with hyaluronidase (100 U/ml, Sigma Aldrich, Cat.-No. H1136-1AMP) for 1 h at 37 °C. Avidin-Biotin blocking was performed before blocking with 10 % 10x TBS, 10 % FBS and 1 % BSA in PBS for 1 h at room temperature. Sections were incubated with a biotinylated HA binding protein antibody (Merck, Cat.-No. 385911-50UG) at 4 °C overnight. After 5 min H_2_O_2_ block, the secondary antibody, horseradish peroxidase conjugated streptavidin (Merck, Cat.-No. OR03L-200UG) was applied to aortic sections for 1 h at room temperature. DAB detection followed by nuclei visualization and microscopy were performed as described for CD44 staining.

### CD44 staining of monocytes

For quantification of CD44 in monocytes, bone marrow monocytes were isolated 7 days post AngII infusion as described above. Five hundred thousand monocytes per chamber (Chamber slides, Nalge Nuc International, NY, USA) were seeded in RPMI medium containing 10 % fetal calf serum (FCS, Gibco, NY, USA) and 100 U/ml penicillin and 100 μg/ml streptomycin (all reagents from Gibco Life Technologies, Paisley, UK) and cultured at 37 °C for 24 h. After incubation, cells were fixed using 3.7 % paraformaldehyde, 5 % glacial acetic acid and 70 % ethanol in PBS for 25 minutes. Cells were washed with PBS and blocked for 1 h with 5 % BSA in PBS and subsequently, incubated at 4 °C overnight with CD44 antibody (1:500, BD Biosciences, Heidelberg, German, #550538) in PBS containing 1 % BSA. The next day, cells were washed with PBS and incubated with a goat anti-rat AF647 antibody (1:400, Gibco Life Technologies, Paisley, UK, #A-21247) for 1 h at room temperature. After washing, cells were mounted with Roti^®^Mount FluorCare DAPI (Roth, Karlsruhe, Germany). Pictures were taken using a Zeiss Axio Observer Z1 microscope (Carl Zeiss Microscopy GmbH, Oberkochen, Germany). Quantification was done using ImageJ software (National Institutes of Health, WI, USA).

### Measurement of soluble CD44

Cardiac blood samples were taken after 7 days of AngII infusion. Cardiac blood was collected into an ethylenediaminetetraacetic acid (EDTA)-coated syringe. The plasma was obtained by two centrifugation steps, first for 15 min at 950xg and then for 5 min at 17,950xg. Plasma was diluted 1:10 in sample diluent buffer before analysis using a mouse CD44 ELISA kit according to the manufacturer's instructions (ab213849, Abcam, Cambridge, UK).

### RNA isolation and quantitative real-time polymerase chain reaction

RNA was isolated from *Apoe*-KO and *Apoe/Has3-*DKO aorta on day 7 post-AngII infusion and primary bone marrow- derived monocytes by using the RNeasy^®^ Plus Universal Kit Mini 50 (Qiagen, Hilden, Germany). RNA concentration and purity (260nm/280nm) were spectrophotometrically determined on a Nanodrop (Peglab, Erlangen, Germany). cDNA was synthesized using the QuantiTect^®^ Reverse Transcription Kit (Qiagen).

QPCR was performed with Platinum SYBR Green qPCR SuperMix-UDG (Life Technologies, Eugene, OR, USA) on a StepOnePlus Real Time PCR System (Applied Biosystems, Carlsbad, USA). Samples were measured in duplicate and relative mRNA expression was calculated using the 2-ΔΔCt method with *Rn18s* as an internal control. Primers used for qPCR are listed in **Supplementary Table 1**.

### RNA-seq analysis of murine aortas

Total RNA samples from DNase-digested aortic tissue were used for transcriptome analyses were quantified (Qubit RNA HS Assay, Thermo Fisher Scientific, MA, USA) and quality measured by capillary electrophoresis using the Fragment Analyzer and the ‘Total RNA Standard Sensitivity Assay’ (Agilent Technologies, Inc. Santa Clara, CA, USA). All samples in this study showed RNA Quality Numbers (RQN) with a mean of 8.7. The library preparation was performed according to the manufacturer’s protocol using the ‘VAHTS™ Stranded mRNA-Seq Library Prep Kit V6’ for Illumina®. Briefly, 500 ng total RNA were used as input for mRNA capturing, fragmentation, the synthesis of cDNA, adapter ligation and library amplification. Bead purified libraries were normalized and finally sequenced on the HiSeq 3000/4000 system (Illumina Inc. San Diego, CA, USA) with a read setup of SR 1x150 bp. The Illumina bcl2fastq tool (v2.20.0.422) was used to convert the bcl files to fastq files as well for adapter trimming and demultiplexing.

Data analyses on fastq files were conducted with CLC Genomics Workbench (version 24.0.1, Qiagen, Venlo, Netherlands). The reads of all probes were adapter trimmed (Illumina TruSeq) and quality trimmed (using the default parameters). Mapping was done against the *Mus musculus* (GRCm39.112; July 17, 2024) genome sequence. After grouping of samples (four/five biological replicates) according to their respective experimental condition, the statistical differential expression was determined using the CLC Differential Expression for RNA-Seq tool (version 2.8, Qiagen, Venlo, Netherlands). The resulting *P*-values were corrected for multiple testing by FDR. A corrected *P*-value of ≤0.05 was considered significant. The CLC Gene Set Enrichment Test (version 1.3, Qiagen, Venlo, Netherlands) was done with default parameters and based on the gene ontology (GO) term ‘biological process’ (*M. musculus*; June 17, 2024). The data was further evaluated with the Ingenuity-Pathway analysis software (Content version: 127006219 (Release Date: 2024-11-17), Qiagen). All data are available in GEO, Accession Nummer GSE305434.

### RNA isolation of murine endothelial cells

The aortas of male *Apoe*-KO and *Apoe/Has3*-DKO mice were removed on day 7 post-AngII infusion, and the abdominal aorta was isolated by cutting just below the diaphragm and just above the common iliac arteries. Vascular rings were cut open and ECs were isolated using an ice-cold metal rod (6 mm diameter) according to the “modified Häutchen method” described by Bruckner et al. (25). Then, RNA from ECs was isolated using the RNeasy Plus micro kit according to the manufacturer`s instructions (Qiagen). To assess RNA quality, the RNA integrity number (RIN) was determined by a 2100 Bioanalyzer (Agilent Technologies, Santa Clara, CA, USA). Only samples with a RIN above 5.0 were processed further.

### RNA-seq analysis of murine endothelial cells

For library preparation, the Trio RNA-Seq Library Preparation kit (TECAN, Männedorf, Switzerland) was used. Five PCR cycles were applied for library amplification and libraries with an average fragment size of 308 bp were sequenced on a NextSeq 550 in paired-end mode (75 bp) at the Genomics & Transcriptomics Labor at the Heinrich Heine University Düsseldorf (Düsseldorf, Germany). For bioinformatic analysis, we used the Galaxy platform (Freiburg Galaxy Project) (26). RNA sequencing reads were mapped using RNA STAR (27) followed by counting reads per gene by using featureCounts (28).

As an additional quality control step the purity of ECs in the respective sample was determined by analyzing expression levels of the classical EC marker genes platelet endothelial cell adhesion molecule (*Pecam1*), von Willebrand factor (*vWF*) and cadherin 5 (*Cdh5*). The normalized counts of these 3 marker genes were added up for each sample and compared with EC marker expression in control adventitial samples from 2 animals (BioProjekt ID: PRJNA1105313). Only samples with EC marker expression of >2-fold of the mean EC marker expression in adventitial samples were included in the analysis. The aortas were not pooled.

In the remaining samples (n=5 for *Apoe*-KO and n=7 for *Apoe/Has3*-DKO), differentially expressed genes (DEGs) were identified by DESeq2 (29). For data visualization and cluster analysis heatmap2 (26) (Freiburg Galaxy Project) was used, for which the gene expression was further normalized using a shifted log transformation [log10 (n+1)].

### Flow cytometry

For the flow cytometric analysis, blood and aortas were harvested 4, 7, and 28 days after implantation of osmotic minipumps. After collecting blood samples by heart puncture erythrocytes were lysed with hypotonic ammonium chloride solution. Aortas were dissected and digested in a solution containing of 1200 U/ml collagenase II (Worthington Biochemicals, Lakewood, NJ, USA), 60 U/ml DNase (Sigma Aldrich, St. Louise, USA) for 60 minutes at 37 °C. After digestion, aortas were filtered through 70 µm cell strainers (Greiner BioOne, Kremsmünster, Austria). Single cell solution was centrifuged at 300 x g for 10 minutes at 4 °C, and resuspended in DMEM (Life Technologies™, Thermo Fischer Scientific, Waltham, MA, USA) supplemented with 1% FCS (Gibco, NY, USA) and incubated at 37 °C for 30 minutes. After second centrifugation step at 300 g for 10 minutes at 4 °C, cell pellets were resuspended in PBS containing 2 mmol/l EDTA and 0.5 % BSA (Sigma Aldrich Steinheim am Albuch, Germany). To avoid unspecific binding, isolated cells were incubated with a CD16/32 antibody, before staining with LIVE/DEAD Fixable Aqua Dead Cell Stain Kit (Thermo Fisher Scientific, Eugene, USA). A list of antibodies including the respective clones, manufacturers, dilutions and definitions of immune cell populations is given in **Supplementary Table 2** and **Supplementary Table 3**.

### Human aortic specimen

Aortic tissue samples were collected and obtained from the local Biobank at the Department of Vascular and Endovascular Surgery at University Hospital Düsseldorf and the Institute of Anatomy Düsseldorf and the Body Donation Program of the HHU. The Ethics Committee of the Medical Faculty of Heinrich-Heine-University Düsseldorf approved the study on human aortic tissue and subjects provided informed consent prior to their participation in the study (patients’ consent) (2018-222_1; 2018-248_1; and 2018-222_7-bio; 2025-3215). The study was conducted in accordance with the Declaration of Helsinki principles and the International Council for Harmonization Guidelines on Good Clinical Practice.

### Statistical analysis

Statistics were performed using the GraphPad Prism 10 Software (La Jolla, CA, USA). If not differently specified, the results are given as mean ± standard deviation (SD). All data sets were tested for outliers using Grubb’s test (α=0.05). To compare the incidence of AD and aneurysm, Fisher’s exact test was performed. The Log-Rank Mantel-Cox test was used to compare survival curves. To compare survival rates between *Apoe-*KO → *Apoe/Has3-*DKO and *Apoe/Has3-*DKO into *Apoe*-KO after BMT experiments, the Gehan-Breslow-Wilcoxon test was used. For continuous variables, normality and homogeneity of variance were assessed by Shapiro-Wilk and Brown-Forsythe tests, respectively. Unpaired *t*-test or Mann-Whitney U-test was used to detect significant differences among 2 groups for normally distributed data or non-normally distributed data, respectively. Multi-group-analyses were made by Kruskal Wallis test followed by Dunn’s test or by One-way ANOVA followed by Tukey’s or Sidak’s multiple comparison test between groups showing normal distribution. For repeated time point measurements, data were analyzed by 2-way ANOVA followed by Tukey’s or Sidak’s *post hoc* tests as appropriate. *P* < 0.05 was considered statistically significant.

## Results

Based on the important role of HA in various inflammatory pathologies, we first asked whether HA content is modified in human aneurysm formation. Indeed, we detected significantly increased amounts of HA in aortic specimens from AAA patients compared to control aortas from subjects without AAA (**Supplementary Figures 1A,B**). Similarly, the protein level of HA receptor CD44 was significantly higher in the human tissue of abdominal aortic aneurysms compared to the control group (**Supplementary Figures 1C,D**).

**Figure 1:**
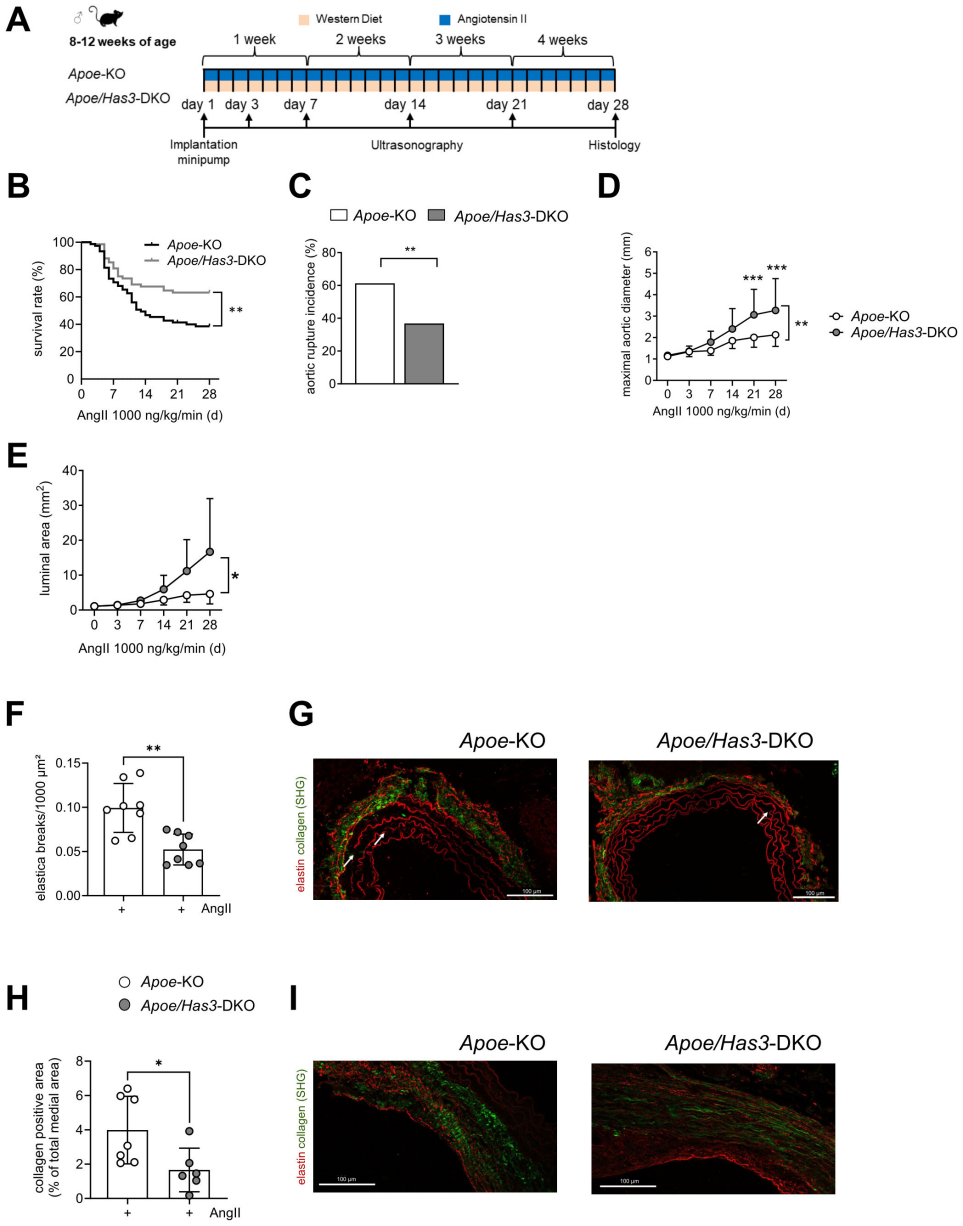
Deficiency in *Has3* protects against aortic ruptures during 28 days of AngII infusion. **(A),** Experimental design: Male *Apoe*-knockout (*Apoe-KO*) and *Apoe/Has3*- double deficient mice (*Apoe/Has3*-DKO) were fed a Western Diet (WD) and infused with AngII (1000 ng/kg/min) for a maximum of four weeks. Ultrasonography was performed at baseline before start of AngII infusion as well as three, seven, 14-, 21- and 28-days post implantation of osmotic minipumps. **(B),** Improved survival in *Apoe/Has3*-DKO mice. Kaplan-Meier curve represents the percentage survival in AngII-infused *Apoe*-KO (n=75) and *Apoe/Has3*-DKO (n=68), *P* = 0.0051, Log-Rank (Mantel-Cox) test. **(C)**, Rupture incidence in *Apoe*-KO (n=75) and *Apoe/Has3*-DKO (n=68), ** *P* < 0.01, Fisher’s exact test. **(D),** Larger maximal aortic diameter and **(E),** luminal area in AngII-infused *Apoe/Has3*-DKO vs. *Apoe*-KO measured by ultrasound; n=14, * *P* < 0.05, ** *P* < 0.01, *** *P* < 0.001. Two-way repeated time-point measures ANOVA followed by Sidak’s *post hoc* test. **(F, G)**, Fewer elastica breaks in aortic aneurysm tissue sections of *Apoe/Has*3-DKO. **(F),** Quantification of the number of ruptures of elastin fibers; n=8, ** *P* < 0.01, unpaired Student’s *t*-test. **G,** Representative elastin staining (red) of aortas of *Apoe-*KO and *Apoe/Has3-*DKO. White arrows indicate breaks of elastic lamella**. (H)**, Reduced collagen deposition in aortas of *Apoe/Has3*-DKO mice; n=7,6, * *P* < 0.05, Mann-Whitney test. Data are presented as means ± SD. **(I)**, Representative sections of the collagen deposition (green, label free) in the suprarenal aortic tissues of *Apoe*-KO and *Apoe/Has3*-DKO mice. SHG: second harmonic generation.

To analyze the role of HA and specifically HAS3-derived HA in AAA in more detail, we used a model of AngII-induced AAA/AD formation and rupture in male *Apoe-* and *Apoe/Has3*-deficient mice fed a Western type diet for a maximum of four weeks (**Figure 1A**).

### Improved survival of *Apoe/Has3*-deficient mice after 28 days of AngII infusion

Mice of both genotypes were fed a Western type diet and received AngII infusion *via* osmotic minipumps (1000ng/kg/min) for four weeks. Radiotelemetry indicated similar blood pressure as well as heart rate values at baseline and the successful induction of hypertension under infusion of AngII. No significant differences between genotypes either during daytime (**Supplementary Figures 2A, B**) or nighttime (**Supplementary Figures 2C, D**) were observed. While after one week of AngII infusion survival rates were similar in both groups, after four weeks, significantly more *Apoe/Has3-*DKO than *Apoe-*KO mice had survived (63.2 % versus 38.7 %) (**Figure 1B**). Upon analyzing the underlying cause, we observed significant differences in aortic rupture incidence between both genotypes: Aortic ruptures were fatal in 46 of 75 *Apoe*-KO mice (61.3 %) whereas only 25 of 68 mice *Apoe/Has3-*DKO mice (36.8 %) succumbed to the same condition (**Figure 1C**). However, *ex vivo* analysis of passive aortic tension revealed no differences in the passive force: Pre-stretched abdominal aorta strips retracted similarly in both *Apoe/Has3*-DKO and the control group at baseline and after 7 days of AngII-infusion (**Supplementary Figures 2E, F**). Additionally, no significant differences were observed between the two strains in body weight changes (**Supplementary Figure 3A****),** cardiac hypertrophy (**Supplementary Figures 3B, C****),** or abdominal aortic peak velocity (**Supplementary Figure 3D**).

While at first glance, in the double-transgenic group, a more prominent enlargement of the suprarenal aorta following 4 weeks of AngII infusion was detected (**Figures 1D, E****;****Supplementary Figures 3E, F**), these findings must be interpreted in the context of higher survival rates of A*poe/Has3-*DKO mice. This clearly leads to a bias when comparing the surviving and probably less affected *Apoe-KO* mice with *Apoe/Has3*-DKO that did not show ruptures.

Histological analysis of AAA sections using SHG microscopy on day 28 revealed significantly less elastic fiber destruction within the aortic media in *Apoe/Has3*-DKO compared with *Apoe*-KO mice, indicating reduced aneurysm pathology (**Figures 1F, G**). Similarly, collagen deposition was significantly lower in the aortas of *Apoe/Has3*-DKO (**Figures 1H, I**).

### *Has3* deficiency attenuates immune cell infiltration and elastica breaks in the aortic wall after one week of AngII infusion

**Figure 2:**
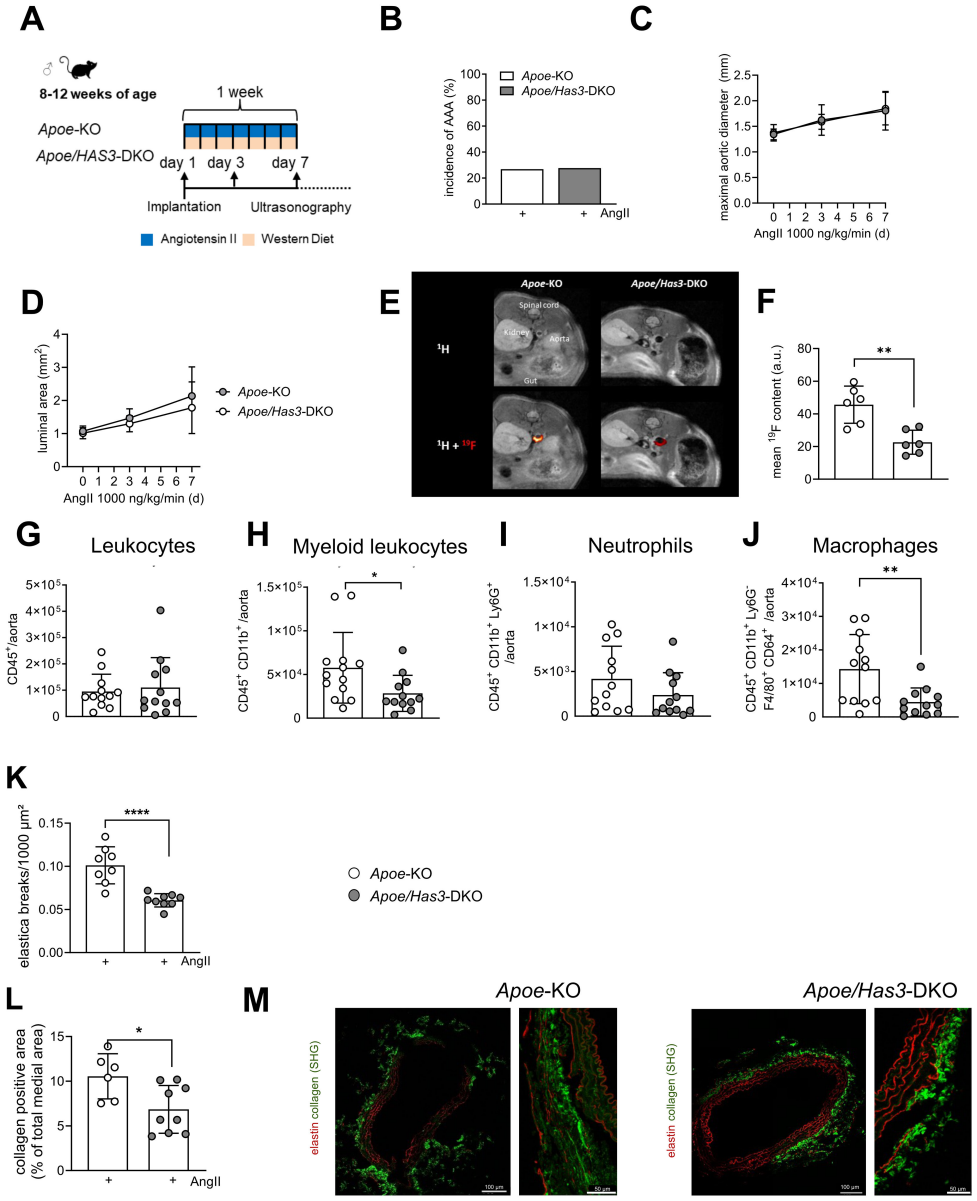
*Has3* deficiency attenuates immune cell infiltration and reduces elastica breaks in the aortic wall after one week of AngII infusion. **(A),** Experimental design: Male *Apoe*-knockout (*Apoe-KO*) and *Apoe/Has3*- double deficient mice (*Apoe/Has3*-DKO) were fed a Western Diet (WD) and infused with AngII (1000 ng/kg/min) for one week. Ultrasonography was performed at baseline before start of AngII infusion as well as three and seven days post implantation of osmotic minipumps. **(B),** Incidence of AAA (estimated as 1.5-fold increase in suprarenal diameter) was not different between *Apoe*-KO and *Apoe/Has3*-DKO after 1 week of the AngII infusion. **(C),** Maximal aortic suprarenal outer diameter and **(D),** luminal area were similar between *Apoe*-KO (n=20) and *Apoe/Has3*-DKOs (n=14), *P* ≥ 0.05, Two-way repeated time-point measures ANOVA. **(E),**^1^H/^19^F MR inflammation imaging in suprarenal aortas of *Apoe*-KO and *Apoe/Has3*-DKO mice. Displayed are axial ^1^H scans of the abdominal area (upper panel) and a merging of ^1^H and the aortic ^19^F signal (hot iron scale; lower panel). **(F)**, Quantification of the ^19^F signal (mean ^19^F signal-to-noise ratio) around the vascular wall (n=6), ** *P* < 0.01, Mann-Whitney test. (**G-J),** No differences in total **(G)**, leukocytes and **(I)**, neutrophils, while fewer **(H),** myeloid leukocytes and **(J),** macrophages accumulate in the aortic wall of *Apoe/Has3*-DKO (n=12) vs. *Apoe*-KO (n=12), * *P* < 0.05, ** *P* < 0.01, unpaired Student’s *t*-test. **(K)**, Decreased number of elastica breaks in AAA from *Apoe/Has3*-DKO (n=9) vs *Apoe*-KO (n=8), **** *P* < 0.0001, Mann-Whitney test. (**L),** Quantification of the collagen positive area, n=6,9, * *P* < 0.05, Mann-Whitney test. (**M)**, Representative sections of the collagen deposition (green, label free) in the suprarenal aortic tissues of *Apoe*-KO and *Apoe/Has3*-DKO. Overview cross-sections of the aorta are shown on the left. To minimize photobleaching after acquisition of 25× SHG images, the higher magnification views of the aortic media shown on the right (**Figure 2M**) were obtained from a different AAA slide. These images are representative of the regions quantified (**Figure 2L**). SHG: second harmonic generation. Data are presented as means ± SD.

Since inflammation is an early hallmark of AAA pathophysiology, we hypothesized that the cause for the beneficial effects of *Has3* deficiency may derive from early changes in the immune response. We therefore undertook a more detailed analysis of AAA development on day 7 after AngII infusion (**Figure 2A**). First, in agreement with our previous results, no differences in body weight (**Supplementary Figure 4A**) or cardiac hypertrophy were observed in *Apoe*-KO and *Apoe/Has3*-DKO mice at this time point (**Supplementary Figures 4B, C**). Further, transthoracic echocardiography revealed similar cardiac function in response to AngII infusion in both groups of mice (**Supplementary Figure 4D**). Incidence of AAA was comparable in both genotypes at this early time point (**Figure 2B**). As expected AngII progressively increased the abdominal aortic diameter, yet no differences between *Apoe*-KO and *Apoe/Has3*-DKO mice were measured at day 7 (**Figure 2C**). Moreover, at this early time point *Has3* deficiency did not affect suprarenal aortic lumen area (**Figure 2D**). However, a significant increase of myeloid leukocyte and monocyte numbers in the peripheral blood of AngII-infused *Apoe/Has3*-DKO mice as compared to *Apoe*-KO was detectable, while lymphocyte levels were preserved (**Supplementary Figure 5**).

To further investigate the inflammatory response to AngII infusion in both groups, we conducted *in vivo*^1^H/^19^F MRI. Five days after osmotic pump implantation, we injected PFCs intravenously to enable ^19^F loading of circulating immune cells. We then used background-free ^19^F MRI to track the labeled cells. To allow sufficient infiltration and accumulation of ^19^F-loaded phagocytic cells (monocytes and macrophages), these investigations were performed 48 hrs later, i.e. after 7 days of AngII infusion. Despite the similar systemic and morphological alterations in the early phase of AngII infusion described above, ^1^H/^19^F MRI surprisingly showed significantly less infiltration of immune cells into the aortic wall of *Apoe/Has3*-DKO compared to *Apoe*-KO mice during this period (**Figures 2E, F**). This finding was confirmed by flow cytometry of aortas demonstrating decreased numbers of both myeloid leukocytes and macrophages in the aortic wall of *Apoe/Has3*-DKO mice (**Figures 2G-J****).** Similar alterations of infiltrating immune cells in the aortic wall were observed in *Has3*-KO mice on a C57BL/6 background, confirming that the impaired recruitment is indeed *Has3*-dependent and not influenced by metabolic changes due to *Apoe*-deficiency (**Supplementary Figures 6A-D**). Interestingly, in contrast to the decreased myeloid cell numbers, increased platelet counts were detected in the aortic wall of *Apoe/Has3*-DKO mice (**Supplementary Figure 7A, B****).**

SHG microscopy demonstrated that the reduced immune cell infiltration into the aortic wall of *Has3*-deficient mice was accompanied by fewer elastica breaks in AAA sections from *Apoe/Has3*-DKO *vs. Apoe*-KO mice after 7 days of AngII infusion (**Figure 2K****).** Moreover, the collagen-positive area was significantly decreased in AAA tissue sections from AngII-infused *Apoe/Has3-*DKO as compared to *Apoe*-KOs mice (**Figures 2L, M**) suggesting also diminished collagen deposition in *Has3* deficiency after 7 days of AngII infusion.

Taken together, the data suggest that the absence of *Has3* in the early phase of AngII infusion results in decreased immune cell infiltration into the aortic wall, which subsequently leads to ameliorated remodeling processes and stabilization of the abdominal aorta, followed by a lower incidence of rupture. Thus, lack of *Has3* results in less initial immune cell accumulation and aortic remodeling which gives rise to an improved survival at day 28 post-AngII infusion.

### Bulk RNA-seq of the aorta indicates altered immune cell dynamics in *Has3*-deficient mice

**Figure 3:**
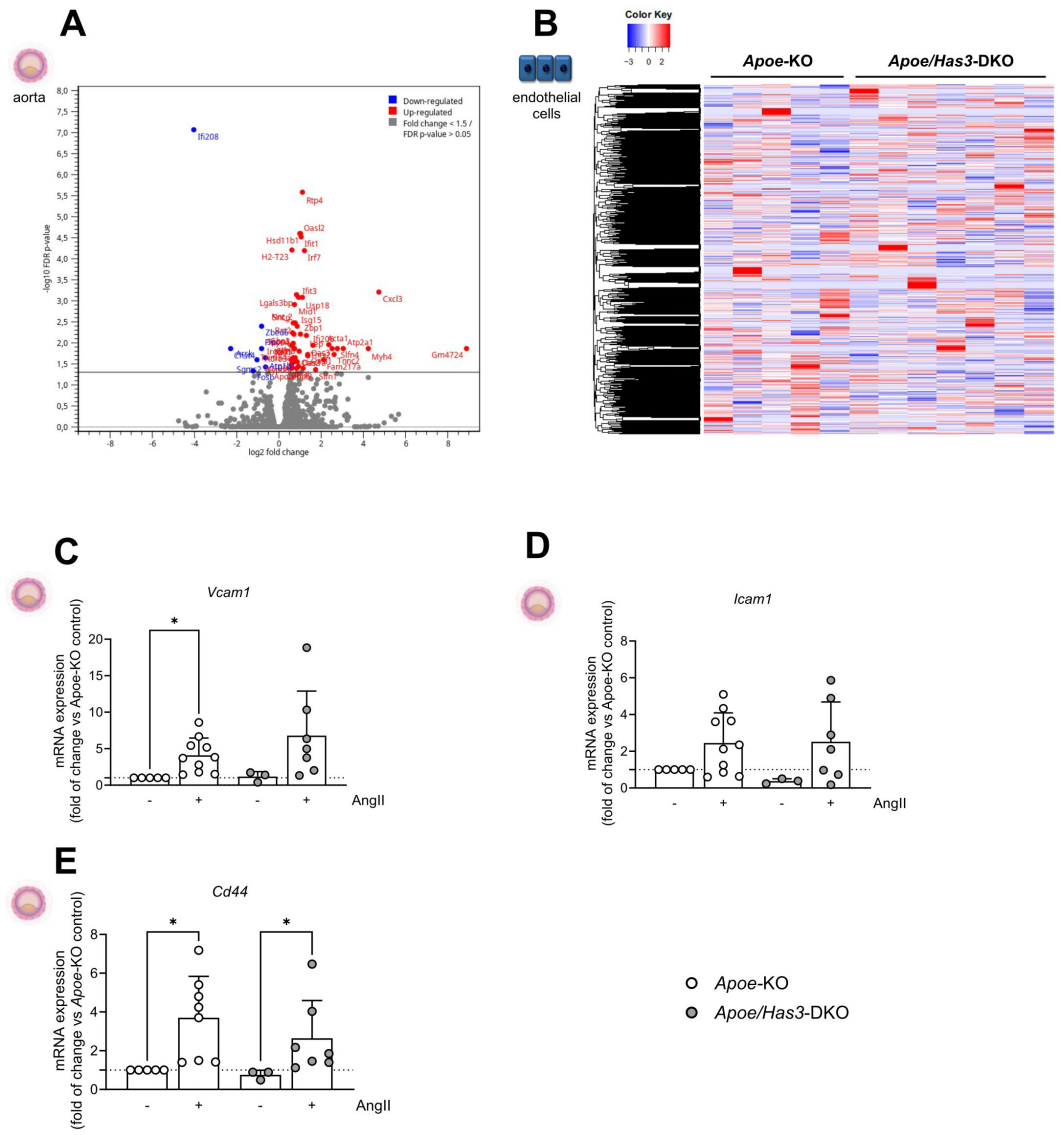
Differentially expressed genes in aortas of *Apoe/Has3*-DKO in the first week of AngII infusion. **(A)**, Volcano-plot presenting the differential gene expression between *Apoe/Has3*-DKO and *Apoe*-KO abdominal aorta identified by bulk RNA-Seq assay. Dots represent the product of log2-transformed fold change and −log10-transformed P values. Eight genes were downregulated (blue) and 45 were upregulated above a fold change of 1.3 in aortas of *Apoe/Has3*-DKO vs *Apoe*-KO using a FDR-corrected *P* value <0.05. **(B)**, RNA-Seq heatmap of expressed genes in endothelial cells derived from the abdominal aorta of Apoe-KO (n=5) and Apoe/Has3-KO (n=7). In red: upregulation, in blue: downregulation. Aortic mRNA expression levels of (**C, D),** adhesion molecules *Vcam1*, *Icam1* (n=5,10,3,7), and **(E),** the receptor for HA *Cd44* (n=5,8,3,7). *P < 0.05, Kruskal-Wallis test followed by Dunn’s multiple comparisons test. Data represent mean ± SD.

To elucidate the underlying mechanism(s) leading to reduced immune cell infiltration into the aortic wall of *Has3*-deficient mice in the early AngII infusion phase, we first carried out bulk RNA-seq analysis of aortas from *Apoe-* and *Apoe/Has3*-DKO mice after 3 days of AngII infusion. This revealed 53 DEGs, of which 8 were downregulated and 45 were upregulated in *Apoe/Has3*-DKO mice (**Figure 3A**). Applying QIAGEN’s Ingenuity® Pathway Analysis tool to our DEGs, “Agranulocyte adhesion and diapedesis” as well as “Granulocyte adhesion and diapedesis” were among the top regulated canonical pathways suggesting an overarching alteration of immune cell trafficking in the absence of *Has3* (**Supplementary Table 4** and **Supplementary Table 5**).

### Gene expression in endothelial cells is similar between genotypes

Based on these results, we investigated whether an alteration of the aortic ECs or infiltrating immune cells play a key role in the altered recruitment into the aortic wall.

For analysis on the cellular level, ECs from the abdominal aorta were isolated using the modified Häutchen method (25). However, subsequent bulk RNA-Seq of the isolated ECs revealed no differences between genotypes (**Figure 3B****).** Most importantly, the genes that were regulated in the whole aorta showed no changes in the isolated ECs (**SupplementaryTable 6**). Accordingly, mRNA expression of *Vcam1* and *Icam1* (mediating leukocyte-EC adhesion) and the receptor for HA *Cd44* were similarly increased by AngII in aortas of *Apoe*-KO and *Apoe/Has3*-DKO mice (**Figures 3C-E****).**

To exclude functional limitations of the ECs, *ex vivo a*ssessment of endothelial function was performed since endothelial dysfunction and impaired NO production may precede AAA formation and progression. However, endothelial function measured as aortic relaxation to acetylcholine-induced endogenous NO production was fully preserved in *Apoe*-KO and *Apoe/Has3*-DKO mice on day 3 post AngII infusion (**Supplementary Figure 8A**). Likewise, aortic smooth muscle relaxation to exogenous NO was similar between the strains (**Supplementary Figure 8B**). While three days of AngII infusion did not affect receptor-independent aortic contraction *via* voltage-operated Ca^2+^ channels (**Supplementary Figure 8C**), a more than doubled aortic contraction in response to alpha1-adrenergic stimulation was observed (**Supplementary Figure 8D**). However, the extent of these changes was again similar in both genotypes. These data indicate the absence of endothelial dysfunction and suggest that *Has3* deficiency does not affect the early functional response of the aortic endothelium and smooth muscle to AngII.

**Figure 4:**
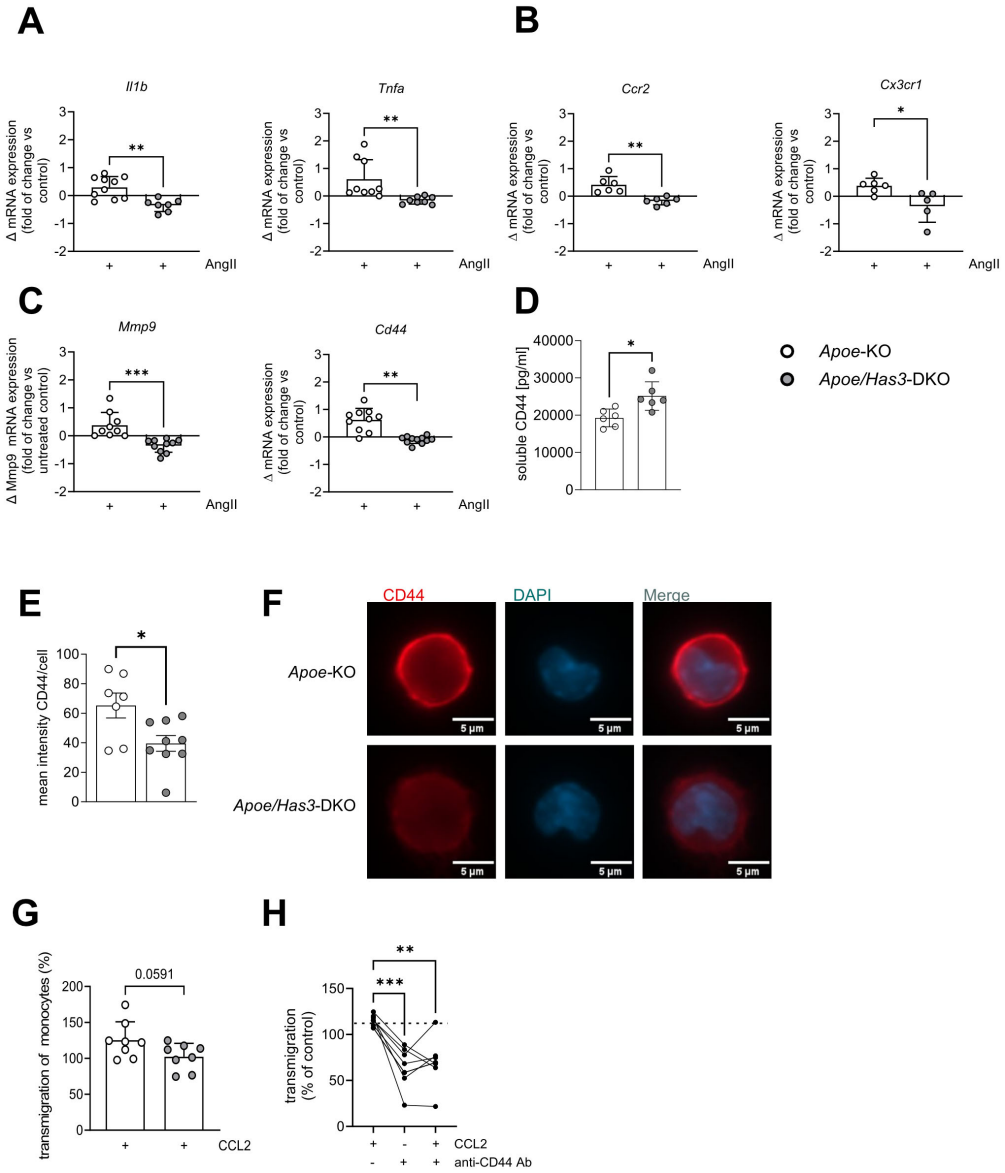
Impaired AngII response and endothelial transmigration in *Has3*-deficient monocytes. **(A-C),** Changes in mRNA expression of **(A),** proinflammatory cytokines *Il1b* (n=10,7) and *Tnfa* (n=9,8), **(B),** chemokine receptors *Ccr2* (n=6) and *Cx3cr1* (n=6,5) and **(C)**, *Mmp9* and the receptor for HA *Cd44 (*n=10,11*)* in bone-marrow derived monocytes of *Apoe*-KO and *Apoe/Has3*-DKO after 6 h of incubation with AngII (10 ng/ml), * *P* < 0.05, ** *P* < 0.01 unpaired Student’s *t*-test or Mann-Whitney test. **(D),** Elevated levels of soluble CD44 in plasma of *Apoe/Has3*-DKO (n=6) as compared to *Apoe*-KO (n=6) on day 7 of AngII infusion. ** *P* < 0.01, Mann-Whitney test. **(E),** Quantification of fluorescence intensity of CD44 staining in monocytes isolated from bone marrow of *Apoe*-KO and *Apoe/Has3-*DKO on day 7 of AngII infusion (n=7,9), * *P* < 0.05, Mann-Whitney test and **(F),** representative images of CD44 (red) and DAPI (blue) co-stainings. **(G)**, CCL2-driven endothelial transmigration of monocytes (expressed as % vs corresponding controls without CCL2) isolated from femurs and tibias of *Apoe*-KO and *Apoe/Has3*-DKO mice (n=8,8) on day 3 post AngII infusion, *P* = 0.059 unpaired Student’s *t*-test. **(H)**, CCL2- induced migration of isolated bone marrow monocytes without (-) or with previous blocking of the HA binding site of CD44 (+) using a specific anti-CD44 antibody (anti-CD44 Ab) or respective IgG control for 24 hours (10 µg/ml). ** *P* < 0.01, Repeated measures one-way ANOVA followed by Šídák's multiple comparisons test. Data are presented as means ± SD.

Next, immune cells were investigated in more detail. Unlike the unchanged endothelial cells, we found mRNA expression of proinflammatory cytokines *Il1b* and *Tnfa* as well as different chemokine receptors such as *Cx3cr1*, *Ccr2* to be upregulated on isolated monocytes of *Apoe*-KO mice in response to AngII while no corresponding upregulation was detected in monocytes of *Apoe/Has3*-DKO mice (**Figures 4A, B**). Importantly, this unresponsiveness to AngII-induced gene expression in the *Has3-*deficient group was also true for *Mmp9* and the HA receptor *Cd44* (**Figure 4C**). Since CD44 regulates immune cell-endothelial interactions and promotes the adhesion of immune cells to the endothelium, we proposed that immune cell recruitment in *Apoe/Has3*-DKO mice might be disturbed. Staining of isolated monocytes from mice of both genotypes for CD44 further confirmed this finding on protein level (**Figures 4E, F**). In addition, increased levels of soluble CD44 were detected in the circulation of *Apoe/Has3*-DKO mice suggesting increased shedding of the receptor (**Figure 4D**). Indeed, we observed a trend towards reduced CCL2-triggered monocyte transmigration *in vitro* (**Figure 4G**). When isolated monocytes were pre-incubated with an anti-CD44 antibody that specifically inhibits HA-dependent signaling, reduced monocyte transmigration was observed, mimicking the phenotype of *Apoe/Has3*-DKO monocytes (**Figure 4H**).

### *Has3* expression in bone marrow-derived cells promotes inflammatory cell infiltration into the aortic wall

**Figure 5:**
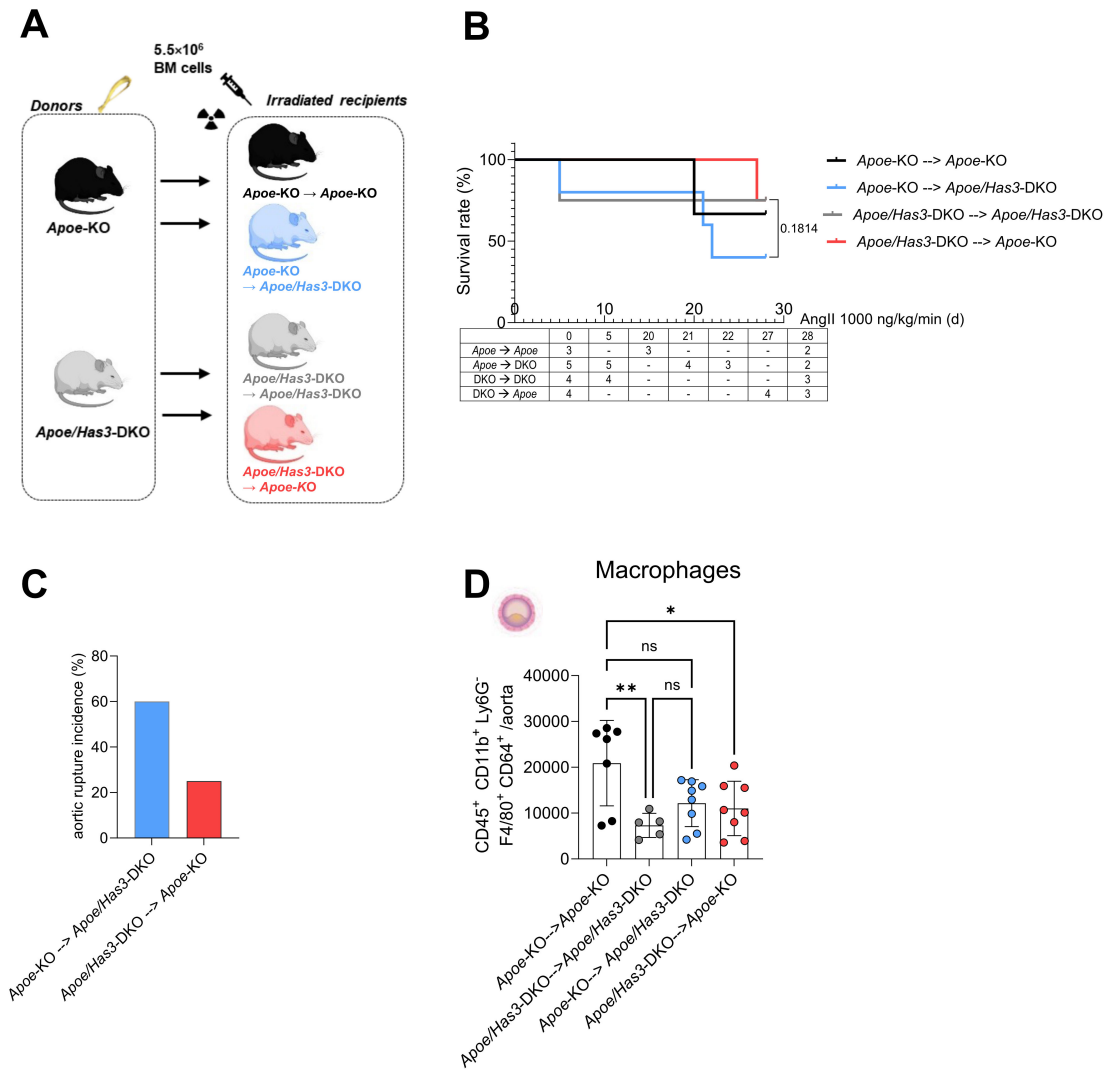
Bone marrow transfer with *Apoe/Has3*-DKO bone marrow provides protection against aortic ruptures and reduces aortic inflammatory cell infiltration after AngII infusion. **(A)**, Experimental outline of bone marrow transfer (BMT). Bone marrow from *Apoe*-KO was transplanted into lethally irradiated (10 Gy) *Apoe*-KO (*Apoe*-KO→*Apoe*-KO), and *Apoe/Has3*-DKO (*Apoe*-KO→*Apoe/Has3*-DKO) mice. Accordingly, BM from *Apoe/Has3*-DKO was transplanted into irradiated *Apoe/Has3*-DKO (*Apoe/Has3*-DKO→*Apoe/Has3*-DKO) and *Apoe*-KO (*Apoe/Has3*-DKO→*Apoe*-KO). Scheme created in https://BioRender.com**(B)**, Survival rates of *Apoe/Has3*-double deficient mice receiving bone marrow from *Apoe-KO* (2/5 surviving animals, blue line, n=5) and *Apoe*-KO that received *Apoe/Has3*-DKO bone marrow (3/4 surviving animals, red line, n=4) and their respective controls (*Apoe*-KO→*Apoe*-KO, 2/3 surviving animals, black line, n=3) and (*Apoe/Has3*-DKO→*Apoe/Has3*-DKO, 3/4 surviving animals, grey line, n=4). Survival analysis includes only animals pre-allocated to 28-day follow-up. Kaplan–Meier survival with numbers at risk (animals alive immediately before each time point) shown below the x-axis. *P* = 0.1814, Gehan-Breslow-Wilcoxon test was used, to compare survival rates in *Apoe/Has3*-DKO→*Apoe*-KO and *Apoe*-KO→*Apoe/Has3*-DKO. **(C)**, Aortic rupture incidence in *Apoe*-KO (25 %, n=4) and *Apoe/Has3*-DKO (60 %, n=5). **(D)**, Levels of macrophages after BMT in the aortic wall of *Apoe-KO* (*Apoe/Has3*-DKO→*Apoe*-KO, n=8) and *Apoe/Has3*-DKO (*Apoe*-KO→*Apoe/Has3*-DKO, n=8) as compared to the corresponding controls (*Apoe*-KO→*Apoe*-KO, n=7, *Apoe/Has3*-DKO→*Apoe/Has3*-DKO, n=5). *P < 0.05, ** *P* < 0.01, One-way ANOVA followed by Sidak’s multiple comparison test. Data represent mean ± SD.

To verify whether immune cells are the key cell type conferring protection against rupture in *Apoe/Has3*-DKO mice, we performed a bone marrow transfer (BMT)/transplantation experiment to distinguish between stromal and bone marrow cell-derived effects *in vivo*. To this end, we lethally irradiated *Apoe*-KO and *Apoe/Has3*-DKO mice and rescued them with bone marrow from *Apoe/Has3*-DKO and *Apoe*-KO donors, respectively (**Figure 5A**). Similar numbers of leukocytes and myeloid leukocytes in the circulation indicated equal reconstitution after BMT in all groups (**Supplementary Figure 9****).** Interestingly, the survival curve of *Apoe/Has3*-double deficient mice receiving bone marrow from *Apoe-KO* exhibited a much steeper decline (**Figure 5B**, blue line) indicating a lower survival rate (2 out of 5 surviving animals) when compared to controls (*Apoe/Has3*-DKO that received *Apoe/Has3*-DKO bone marrow, **Figure 5B**, grey line, 3 out of 4 surviving animals) or *Apoe-*KO receiving *Apoe-*KO bone marrow (**Figure 5B**, black line, 2 out of 3 surviving animals). Thus, transplantation of bone marrow from *Apoe* mice largely eliminated the protective phenotype of *Apoe/Has3-*DKO mice. In contrast, transplanting *Apoe/Has3*-DKO bone marrow in *Apoe*-KO recipients seemed to rescue survival (**Figure 5 B**, red line, 3 out of 4 surviving mice, Gehan-Breslow-Wilcoxon test, P = 0.1814) thus pointing towards a crucial role of bone marrow-derived cells expressing HAS3 in impaired survival. Indeed, rupture rates were drastically reduced in *Apoe*-KO recipients of *Apoe/Has3*-DKO bone marrow (**Figure 5C**, red bar, 3 out of 5 mice with aortic ruptures in *Apoe/Has3*-DKO that received *Apoe-*KO bone marrow compared to 1 out of 4 *Apoe*-KO mice with aortic ruptures after receiving *Apoe/Has3*-DKO bone marrow). Moreover, *Apoe*-KO mice that received bone marrow cells from *Apoe/Has3-*DKO (**Figure 5D**, red circles) showed a significant decrease of immune cell recruitment to the aortic wall, while *Apoe/Has3-*DKO receiving bone marrow cells from *Apoe*-KO mice (**Figure 5D**, blue circles) showed a 66 % increase (*P* = 0.08) in monocyte recruitment to the aortic wall both compared to their respective controls (**Figure 5D***Apoe*-KO→*Apoe*-KO, black circles and *Apoe/Has3*-DKO→*Apoe/Has3*-DKO, grey circles).

**Figure 6:**
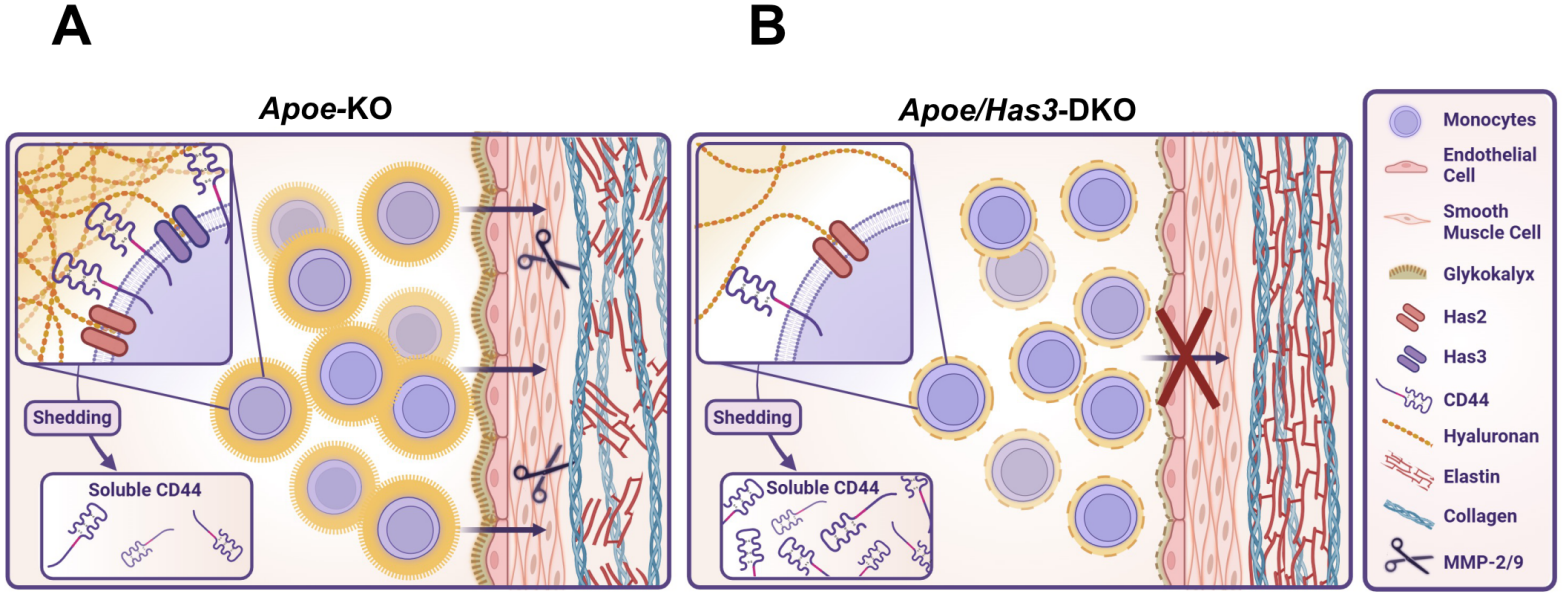
Graphical abstract. **(A),** Induction of AngII-induced AAA/AD in *Apoe*-deficient mice promotes the recruitment of monocytes to the vascular wall. Increased amounts of MMP2 and 9 drive the degradation of elastic fibres thus increasing the risk of aortic ruptures. **(B),** In *Apoe/Has3*-defciency, reduction of the monocytic HA-rich pericellular coat decreased CD44 surface expression and diminishes HA-CD44 mediated immune cell infiltration. Finally, this results in attenuated degradation of the aortic wall and ultimately less ruptures of the aortic wall. Created in https://BioRender.com.

In summary, these data suggest that innate immune cells, in particular monocytes/macrophages, rather than stromal cells account for the beneficial effects of *Has3* deficiency in our model of AAA: Due to reduced HAS3-mediated HA-CD44 immune cell infiltration and the subsequent attenuated degradation of the aortic wall, there is ultimately less destabilization and rupture of the vessel wall (**Figure 6****).**

## Discussion

In the present study, we investigated the role of HAS3-derived HA in the development and progression of AAA. In a murine model of AngII-induced AAA/AD, we demonstrate that genetic deletion of *Has3* results in significantly decreased aortic ruptures and improved survival rates. This was associated with reduced numbers of elastica breaks in the aorta and reduced infiltration of leukocytes, mostly monocytes, into the vessel wall of double-transgenic mice. While ECs could be excluded as drivers of this process, monocytes from *Has3*-deficient mice exhibited decreased AngII-induced polarization towards a proinflammatory and matrix-degrading phenotype. Furthermore, monocytes had reduced expression of adhesion markers known to be crucial for immune cell adhesion and diapedesis. Most importantly, a strong downregulation of the HA receptor CD44 was observed in monocytes when HAS3 was missing, thereby impairing HA-immune cell interactions and contributing to reduced aortic immune cell infiltration (**Figure 6**).

Aortic aneurysm formation and progression has been reported to be driven by three major factors: matrix remodeling and degradation (30), inflammatory responses (31) as well SMC apoptosis and phenotype switches (32). Of note, HA as a major component of the vascular ECM has been described to be involved in all these processes. In the present study aortic RNA bulk sequencing gave no evidence for specific alterations in SMC-related genes. Instead, the underlying pathomechanism was related to an altered immune cell response, specifically alterations in cell movement, adhesion and diapedesis. Indeed, HA has been reported to contribute to immune cell adhesion, e.g. in inflammatory bowel diseases *via* formation of HA-cables (33). Similar structures were also observed in vascular SMCs where HA and its binding molecules serve as an adhesive matrix for T cells, monocytes and macrophages (34).

The role of specific HA-synthesizing isoenzymes in this context is still incompletely explored. It is assumed that the various HA-synthesizing isoenzymes have specific functions and are therefore not necessarily interchangeable in their function. An important role in the inflammatory context was described for HAS3 and previous work highlighted that this isoenzyme especially plays a crucial role in a variety of inflammatory conditions such as experimental neointimal hyperplasia (9), or arteriogenesis (10). However, in these diseases the phenotype is driven by direct effects of HAS3 on vascular SMCs (9) or ECs (10, 11) resulting in altered SMC activation/migration and EC-mediated leukocyte recruitment. The changes in the immune cells are therefore secondary to the changes in the SMCs and ECs.

However, the phenotype described here, is different: *Has3* deficiency did not change AngII-induced hypertension, aortic endothelial function and hypercontractility at the initial phase of AAA/AD development. These results together with the lack of differences in gene expression between ECs isolated from *Apoe/Has3*-DKO and *Apoe*-KO, do not support the involvement of HAS3/HA-dependent changes in ECs or in SMCs in AAA/AD at the investigated early stage of pathogenesis. In contrast, we here report for the first time functional effects of HAS3 deficiency on monocytes leading to a decreased responsiveness in the AngII model of AAA/AD. We show that lack of HAS3 strongly dampens the inflammatory phenotype and migratory capacity of monocytes. This is most evident by the impaired AngII-induced increase in proinflammatory cytokines and chemotactic receptors. In fact, we observed marked absence of AngII-induced upregulation of receptors for cell migration and adhesion to the endothelium such as CX3CR1 and CCR2 in *Apoe/Has3*-DKO mice. Also, aortic bulk RNA seq analysis pointed to a modulation of pathways that influence (a)granulocyte adhesion and diapedesis. Since many of the involved genes were unexpectedly upregulated in the aortic wall in *Apoe/Has3*-deficient mice, this increase in parallel with decreased immune cell invasion could be interpreted as a compensatory mechanism in the sense that this upregulation attempts to compensate for a defect in immune cell infiltration. Of note, gene expression in the aorta did not indicate any effect of *Has3* deficiency on vascular smooth muscle cell function. This contrasts with a model of atherosclerosis, where *Has3* loss led to an ECM-producing SMC phenotype - replicated *in vitro* by CD44 blocking - and was linked to increased plaque stability (35). However, as numerous studies have shown, HAS3 function is highly disease-dependent.

Consistent with the reduced aortic immune cell infiltration observed in *Has3*-deficient mice, we detected lower expression of the chemokine receptors CCR2 and CX3CR1 in monocytes. Both receptors play a crucial role in monocyte recruitment, as demonstrated in conditions such as atherosclerosis (36), kidney injury (37), and pulmonary hypertension (38). Their downregulation is also associated with a less proinflammatory phenotype (39) in line with our finding of reduced TNFα and IL-1β expression. Further, they are the major drivers of Ly6C^high^ monocyte infiltration in atherosclerotic lesions. While in AAA, the CCL2/CCR2 axis has been shown to drive AA formation, the role of CX3CR1 is much less studied. An accumulation of CX3CR1-expressing cells was detected in AngII-infused mice (13, 39).

Thus, the monocytic HA-rich matrix shapes the functional phenotype of the cells and regulates immune cell effector functions under inflammatory conditions induced by AngII. This is in line with a previous study showing that isolated bone-marrow-derived monocytes not only express all three HAS isoenzymes but also that pericellular HA protects the cells against apoptosis (24).

We identify the HAS3-derived HA–CD44 axis as a likely mechanistic pathway driving monocyte infiltration and vascular wall degradation. Similar to findings in T cells after myocardial ischemia/reperfusion (I/R) (12), monocytes isolated from *Has3*-deficient mice with AAA displayed reduced CD44 surface expression and impaired migration across an endothelial layer. One explanation, also proposed in the context of I/R (12), is that pericellular HA protects CD44 from proteolytic shedding by ADAM10, ADAM17, and membrane-bound MMPs (40). Supporting this, we observed elevated circulating levels of soluble CD44 in *Has3/Apoe*-DKO mice.

The importance of CD44 in immune cell recruitment has been demonstrated across multiple pathologies and leukocyte subsets (41). For example, global *Cd44* deletion prevented thoracic aortic dissection by limiting neutrophil infiltration and migration through the endothelium (21). Consistently, our findings in *Has3*-deficient monocytes confirm that monocytic HAS3-derived HA and CD44 are critical for immune cell invasion and AAA pathogenesis (12, 21).

Contrary to monocytes, elevated platelet numbers were found in the aortas of *Apoe/Has3*-DKO mice. This is notable since a recent study suggests that early platelet accumulation in AAA may stabilize the aortic wall and protect against rupture (42). Mechanistically, CD44 may again be involved, as *Cd44*-deficient platelets display enhanced P-selectin and integrin activation (43). Such compensatory upregulation of adhesion molecules could explain the increased aortic platelet accumulation in our model, assuming that *Has3* deficiency similarly reduces CD44 surface expression in platelets. However, investigating the platelet phenotype was beyond the scope of this study.

Finally, potential effects on endothelial CD44 provide an additional layer of regulation. Despite similar endothelial gene expression between genotypes, loss of *Has3* could reduce glycocalyx HA, limiting CD44 protein expression and impairing monocyte arrest. Moreover, endothelial CD44v3, which binds chemokines such as CCL5 and CXCL12 *via* heparan sulphate chains, may be less effective in presenting chemokines to monocytes, thereby weakening recruitment signals (34, 44). Associated with reduced monocyte infiltration into the aortic wall, reduced elastic fiber breaks were observed in *Apoe/Has3*-DKO mice compared to *Apoe*-KO controls. The loss of elastic fibers is a key feature of aneurysm development, with elastin content continuously decreasing during aneurysm growth (45). In this context, MMPs are major drivers of ECM destruction in aortic aneurysm formation (46). Since elastic fibers are preferentially degraded by MMP-2, MMP-9, and MMP-12, they have been extensively studied in AAA. Considering now the properties of the HA-rich matrix with regard to the binding and regulation of cytokines, chemokines and proteases (47), the absence of the HA-rich matrix may also be a factor here. In our study, AngII increased mRNA expression of *Mmp9* in isolated monocytes from *Apoe*-KO mice while this upregulation was abolished in *Apoe/Has3*-DKO littermates *(*48*)* Moreover, the association between reduced *Mmp9* and reduced monocytes in the aortas of *Has3*-deficient mice strongly suggests monocytes as a major MMP-9 source and completes the picture of the underlying mechanisms leading to diminished elastin fragmentation in these mice. Indeed, previous studies showed that inflammatory cell-derived MMP-9 drives AAA formation and degradation of elastic lamellae (49). In line, disruption of elastic lamellae was decreased in mice receiving anti-inflammatory M2 macrophages with decreased amounts of active MMP-2 and MMP-9 (50). Therefore, the observed phenotype of reduced activation of monocytes by AngII matches the reduced incidence of elastic fiber fragmentation and subsequent aortic ruptures in *Apoe/Has3*-DKO mice.

In conclusion, the complex pathomechanisms involving HAS3-mediated effects on monocyte recruitment and function result in reduced rupture incidence in *Apoe/Has3*-DKO mice. We believe that our data are of significant translational relevance, in particular since we provide evidence for increased HA accumulation and CD44 expression in human AAA. The identification of this novel regulatory role for HAS3 may provide the basis for the development of new strategies to prevent AAA formation and rupture.

## Data Availability

The RNA sequencing datasets presented in this study can be found in online repositories. The names of repositories and accession number(s) can be found in the article/**Supplementary Material**.

## Glossary

AAA: abdominal aortic aneurysm
AD: aortic dissection
*Apoe*: apolipoprotein E
*Apoe*-KO: Apoe knockout mice
*Apoe/Has3*-DKO: Apoe/Has3 double knockout mice
AngII: angiotensin II
BAPN: β-Aminopropionitrile
BDM: butanedione monoxime
BMT: bone marrow transfer
BSA: bovine serum albumin
CCR2: C-C chemokine receptor type 2
CCL2: chemokine (C-C motif) ligand 2
CCL5: chemokine (C-C motif) ligand 5
CD44: cluster of differentiation 44
Cdh5: cadherin 5
CX3CR1: CX3C motif chemokine receptor 1
CXCL12: CXC Motif Chemokine Ligand 12
DAB: 3,3'-Diaminobenzidine
DAPI: 4′, 6-Diamidine-2′-phenylindole dihydrochloride
DEG: differentially expressed gene
DMEM: Dulbecco's Modified Eagle Medium
DTT: dithiothreitol
EC: endothelial cell
ECM: extracellular matrix
EDTA: ethylenediaminetetraacetic acid
FCS: fetal calf serum
FBS: fetal bovine serum
FOV: field of view
GO: gene ontology
HA: hyaluronan
HAS: hyaluronan synthase
HEPES: 4-(2-hydroxyethyl)-1-piperazineethanesulfonic acid
I/R: ischemia/reperfusion
MMP: matrix metalloproteinase
MRI: magnetic resonance imaging
NMR: nuclear magnetic resonance
PBS: phosphate buffered saline
Pecam1: platelet endothelial cell adhesion molecule
PFA: paraformaldehyde
PFCs: perfluorocarbon nanoemulsions
PW: pulsed wave
qPCR: quantitative real-time PCR
RARE: rapid acquisition and relaxation enhancement sequence
RIN: RNA integrity number
RHAMM: receptor of HA-mediated motility
RPMI: Roswell Park Memorial Institute (RPMI) medium
RQN: RNA Quality Numbers
SHG: second harmonic generation
