# Supplementary material for: Hyaluronan synthase 3 deficiency lowers the incidence of ruptures of abdominal aortic aneurysms by reducing monocyte infiltration
Source: Front Immunol. 2025 Nov 6;16:1680246. doi: 10.3389/fimmu.2025.1680246 (PMC12631607; doi:10.3389/fimmu.2025.1680246)
Supplement: Supplementary file 1 [file DataSheet1.docx]

Supplementary Material

## Supplementary Figures

##
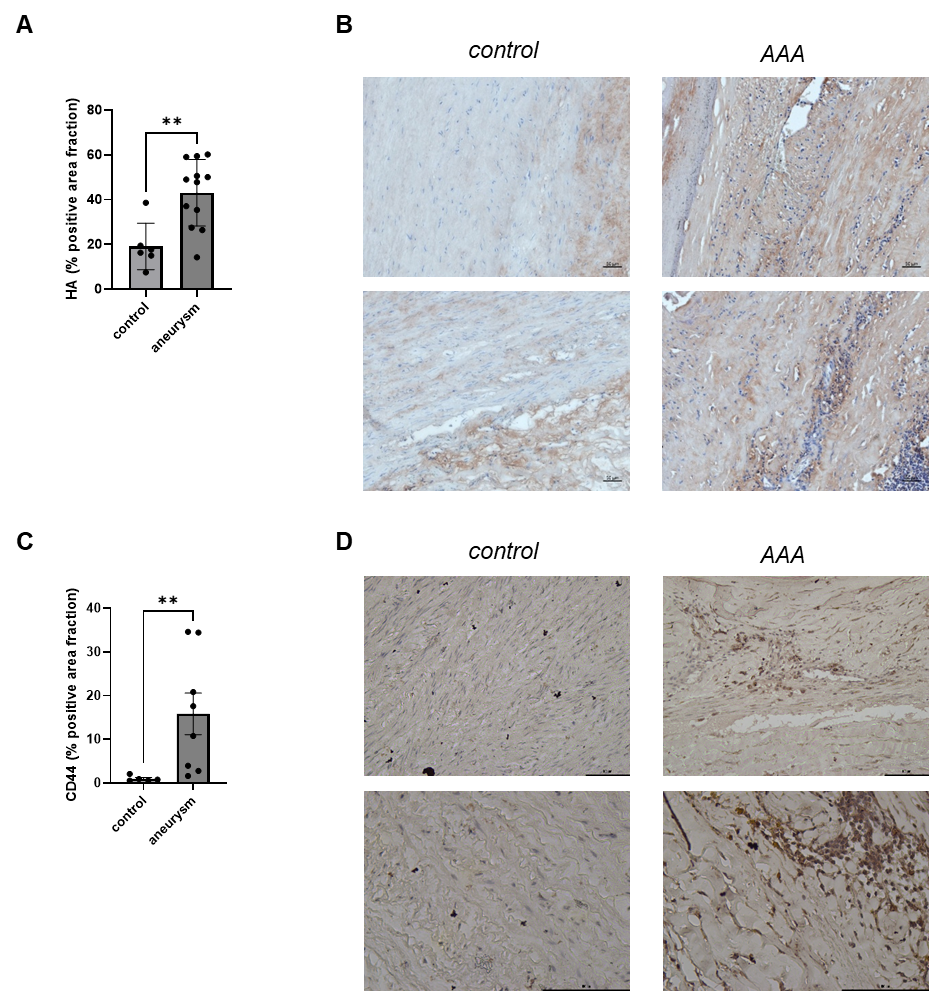


**Supplementary Figure 1.** **Increased hyaluronan accumulation and CD44 protein expression in human aortic aneurysm specimens.**

**A,B,** Staining of hyaluronan (HA)-binding protein and in aortic tissue from individuals without (control, n=6) or with (n=12) abdominal aortic aneurysm. Scale bars: 50 µm. ** P<0.01, unpaired t-test. Data are represented as mean ± SEM. **C,D,** Staining of CD44 in aortic tissue from individuals without (control, n=5) or with (n=8) abdominal aortic aneurysm. Scale bars: 100 µm (D, upper panel) and 50 µm (D, lower panel). ** P<0.01, Mann-Whitney test. Data are represented as mean ± SEM.


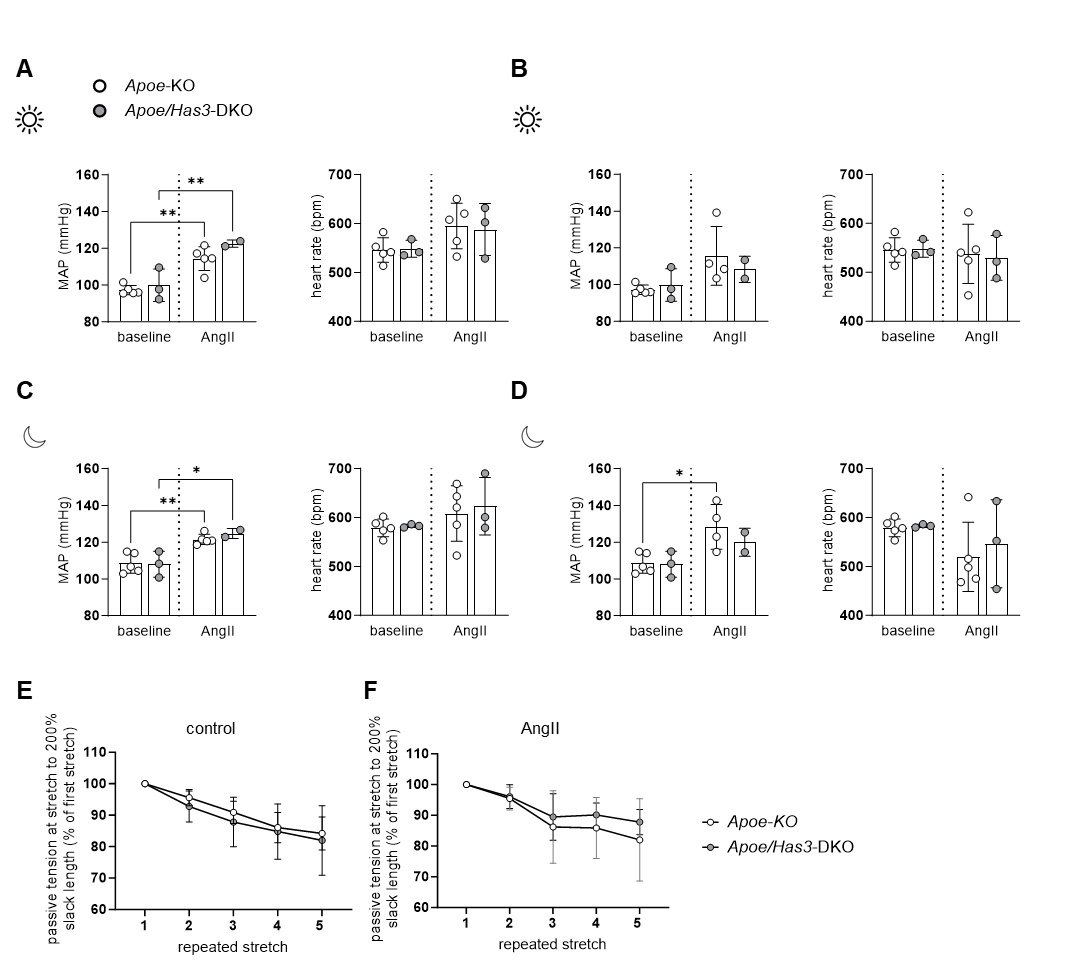


**Supplementary Figure 2.** **No effect of Has3 deficiency on mean arterial blood pressure (MAP) and aortic passive tension after AngII infusion.**

​**A,B** Diurnal and **C,D**, nocturnal MAP and heart rate after **A, C** three days and **B, D** one week of AngII infusion in *Apoe*-KO (n=5) and *Apoe/Has*3-DKO (n=3,2) mice. (One *Apoe/Has3-*DKO mouse died on day 3 of the AngII infusion and in one *Apoe/Has3*-DKO mouse the blood pressure values were not detected while heart rate was properly acquired). ***** *P* < 0.05, ****** *P* < 0.01 vs. baseline, Two-way ANOVA repeated time-point measures Sidak’s post hoc test. Data are presented as means ± SD**. E,F** Passive tension decline after repeated stretch to 200% of slack length in *Apoe*-KO and *Apoe/Has3*-DKO aortic strips in **E**, control and **F**, AngII-infused mice on day 7 post-infusion. In aortic strips from control (n=3,3) and AngII-infused (n=5,4) *Apoe*-KO and *Apoe/Has3*-DKO mice, passive tension values decreased with each repetition while no significant differences between the genotypes were detected. Data were analyzed using two-way ANOVA and are presented as means± SD.

**
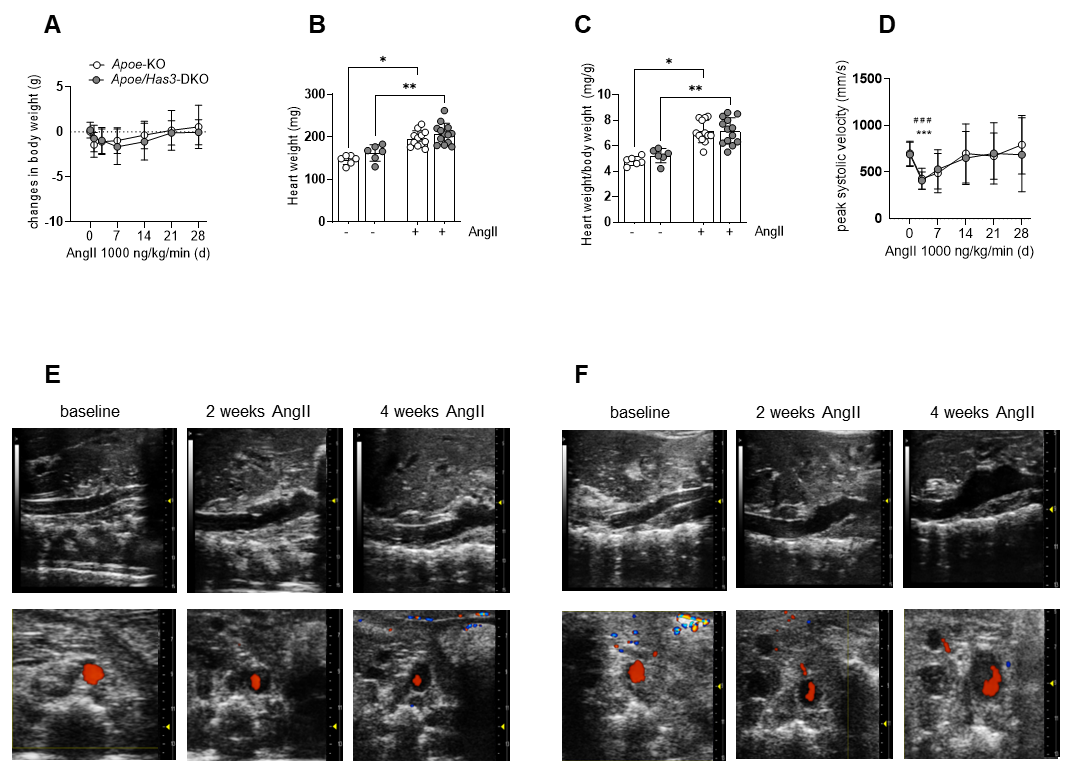
**

**Supplementary Figure 3.** **Cardiac hypotrophy and time course of AAA development in *Apoe*-KO and *Apoe/Has3*-DKO mice infused with AngII for 28 days.**

**A,** Body weight changes (n=12,13), Two-way repeated time-point measures ANOVA, **B,** heart weight and **C,** heart weight to body weight ratios in placebo- and AngII-infused *Apoe*-KO and *Apoe/Has3*-DKO mice (n=6,6,12,13), **P* < 0.05, ***P* < 0.01, Kruskal-Wallis test followed by Dunn’s multiple comparisons test. **D,** Peak systolic blood flow velocity in the suprarenal aorta of AngII-treated *Apoe*-KO (n=11) and *Apoe/Has3*-DKO (n=12),*** *P* < 0.001 and ^###^ *P* < 0.001 vs. baseline for *Apoe*-KO and *Apoe/Has3*-KO accordingly, Two-way ANOVA followed by Sidak’s post hoc test. Data are presented as means ± SD. **E,F,** Representative longitudinal images showing an increase of the aortic diameter (upper panel) and B-mode visualization of the lumen extension and the suprarenal flow with color flow Doppler (red, lower transverse panel) in **E,** *Apoe*-KO and **F,** *Apoe/Has3*-DKO at baseline and 2 and 4 weeks after AngII infusion.


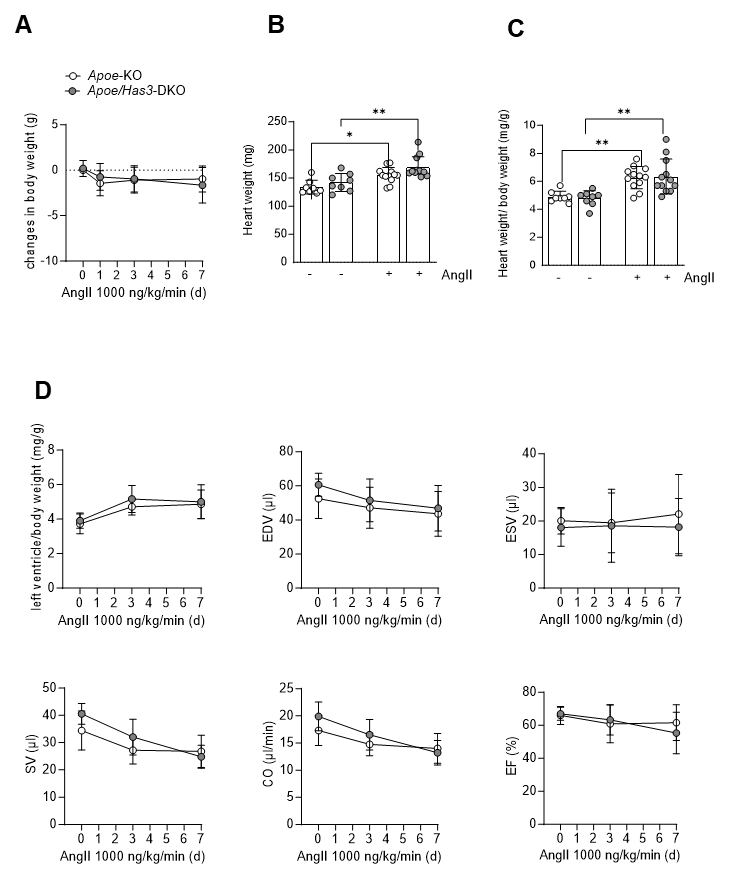


**Supplementary Figure 4.** ***Has3* deficiency does not affect cardiac function at baseline and after one week of AngII infusion.**

**A,** Body weight changes in *Apoe*-KO (n=12) and *Apoe/Has3*-DKO mice (n=13), Two-way repeated time-point measures ANOVA. **B,** Heart weight and **C,** heart weight to body weight ratios in placebo- and AngII-infused *Apoe*-KO and *Apoe/Has3*-DKO mice, (n=8,8,12,12). *P < 0.05, **P < 0.01, One way ANOVA followed by Tukey’s multiple comparisons test. **D,** Echocardiographic analysis revealed identical left ventricle hypertrophy and cardiac response to AngII infusion in *Apoe*-KO (n=7) and *Apoe/Has3*-DKO (n=7), *P* ≥ 0.05, Two-way repeated time-point measures ANOVA. Data are presented as means ± SD. ESV; end-systolic volume, EDV; end-diastolic volume, SV; stroke volume, CO; cardiac output, EF; ejection fraction.


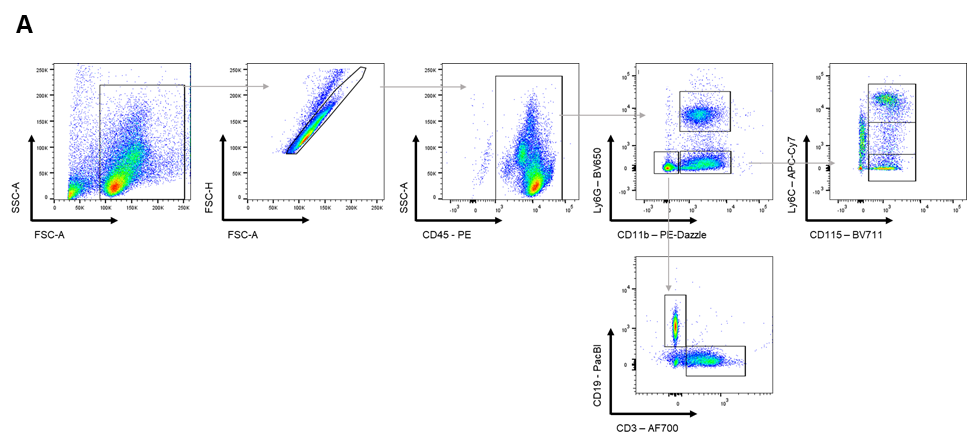

**Supplementary Figure 5. Similar levels of lymphocytes and an increase of myeloid leukocytes and monocytes in the peripheral blood of *Apoe/Has3*-DKO as compared to *Apoe*-KO mice on day 7 after AngII infusion.**

**A**, Gating strategy to identify circulating immune cells by flow cytometry. **B-J,** Flow cytometric analysis of the peripheral blood revealed no significant difference in **B**, leukocytes, **D,** neutrophils, **F**, Ly6C^high^ and **G,** Ly6C^low^ monocytes,^,^ **H,** lymphocytes, **I,** B cells and **J,** T cells between *Apoe/Has3*-DKO (n=7) and *Apoe*-KO (n=18) one week after AngII infusion (P ≥ 0.05, Mann-Whitney test), while **C,** myeloid leukocytes and **E,** monocytes were significantly increased in *Apoe/Has3*-DKO mice, *P < 0.05, Mann-Whitney test. Data are presented as means ± SD.

**Supplementary Figure 6. *Has3* deficiency reduces aortic immune infiltration after one week of AngII infusion and BAPN administration independent of *Apoe-*deficiency.**

No differences in total **A**, leukocytes and **B**, myeloid leukocytes, and **C,** neutrophils, while fewer **D,** macrophages accumulate in the aortic wall of *Has3*-KO (n=6) vs. *Has3*-WT (n=8), * *P* < 0.05, unpaired Student’s *t*-test.


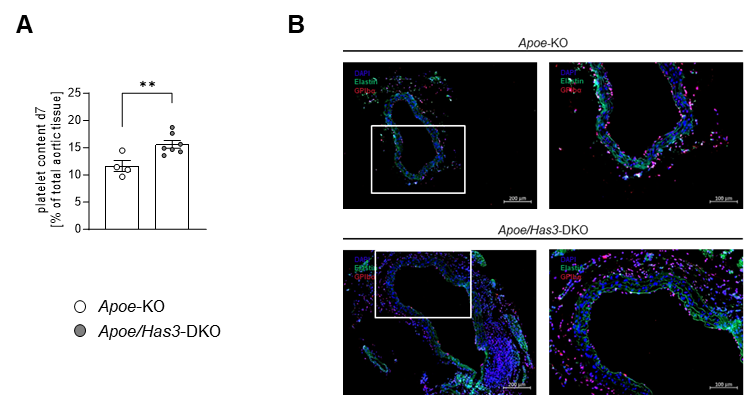


**Supplementary Figure 7. Platelet accumulation in the aortic wall of *Apoe/Has3*-DKO mice on day 7 of AngII infusion**.

**A**, Quantification and **B**, representative immunofluorescence images of platelets into the aneurysm segments of *Apoe*-KO and *Apoe/Has3*-DKO mice (n=4,7). Aortic tissue was specifically stained for platelets (anti-GPIbα/Cy5; red). Elastin autofluorescence is shown in green, nuclei were stained with DAPI (blue). Scale bars: 100 µm (zoom in) and 200 µm (overview).  ** *P*<0.01, Mann-Whitney test. Data are presented as means ± SEM.


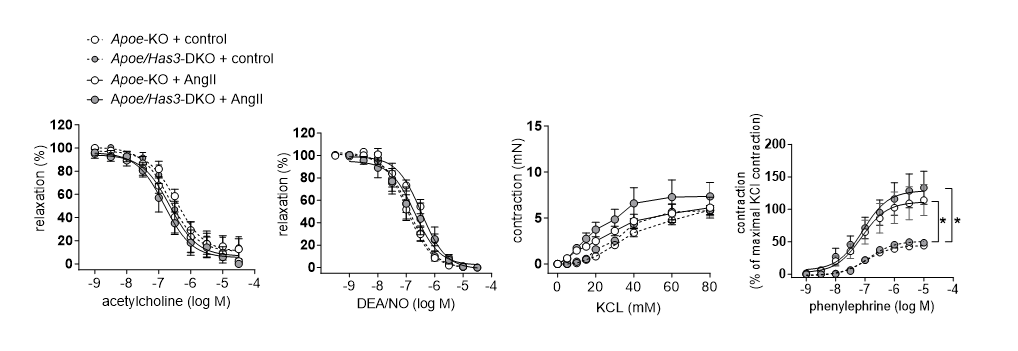


**B**

**C**

**D**

**A**

**Supplementary Figure 8. No effect of *Has3* deficiency on *ex vivo* aortic wall reactivity at baseline and after 3 days of AngII treatment.**

Aortic reactivity to **A**, acetylcholine, **B**, the spontaneous NO donor DEA/NO, **C**, potassium chloride and **D**, phenylephrine as obtained by concentration-response curves in isolated aortic rings of placebo- (n=4) and AngII-infused (n=4,5) *Apoe-*KO and *Apoe/Has3*-DKO mice. * *P* < 0.05, Two-way repeated time-point measures ANOVA. Data are mean ± SEM.


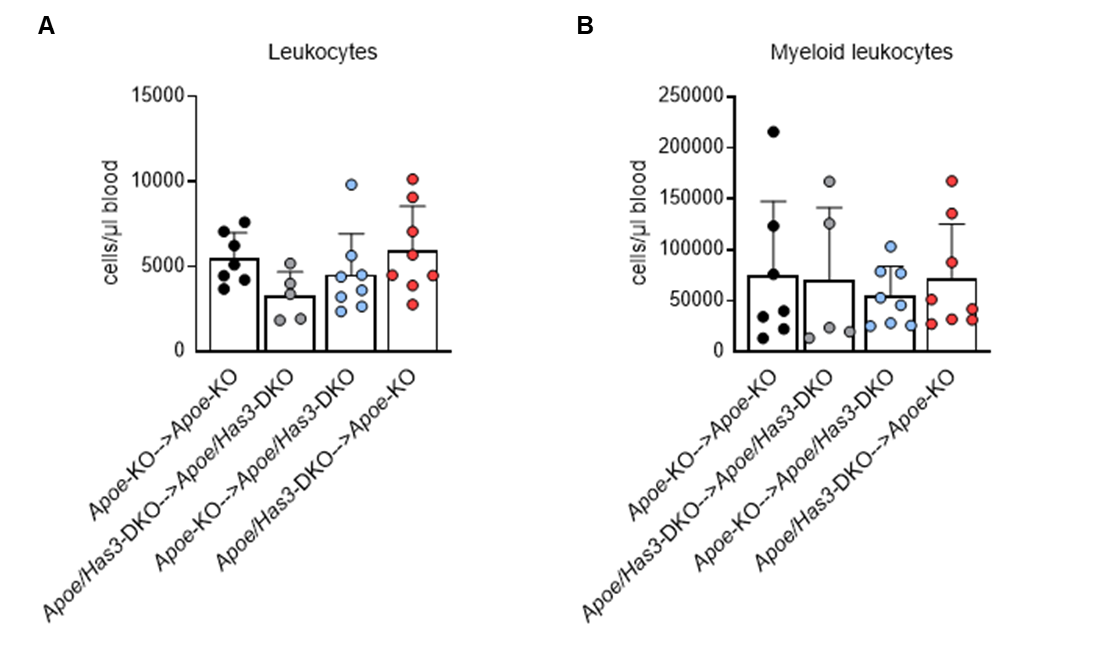


**Supplementary Figure 9. Equal reconstitution in *Apoe*-KO and *Apoe/Has3*-DKO mice after bone marrow transfer.**

Levels of circulating leukocytes and myeloid leukocytes after BMT in the blood of *Apoe*-KO (*Apoe/Has3*-DKO→*Apoe*-KO, n=8) and *Apoe/Has3*-DKO (*Apoe*-KO→*Apoe/Has3*-DKO, n=8) as compared to the corresponding controls (*Apoe*-KO→*Apoe*-KO, n=7, *Apoe/Has3*-DKO→*Apoe/Has3*-DKO, n=5). One-way ANOVA followed by Sidak’s multiple comparison test. Data represent mean + SD.

## Supplementary Tables

**Supplementary Table 1. Murine primer sequences used for qPCR.**

| **Gene** | **Forward** | **Reverse** |
| --- | --- | --- |
| **18S** | GCAATTATTCCCCATGAACG | GGCCTCACTAAACCATCCAA |
| **Cd44** | GACCGGTTACCATAACTATTGTC | CATCGATGTCTTCTTGGTGTG |
| **Mmp9** | CCTGAAAACCTCCAACCTCA | GCTTCTCTCCCATCATCTGG |
| **Tnf alpha** | TCGAGTGACAAGCCTGTAGC | AAGGTACAACCCATCGGCTG |
| **Il1 beta** | GGATGAGGACATGAGCACCT | CGTCACACACCAGCAGGTTA |
| **Ccr5** | AGACATCCGTTCCCCCTACA | GCAGGGTGCTGACATACCAT |
| **Cx3cr1** | AGTGTGTCGGGTGTCCATTC | GGTAAGGCGAGTCAGCAGTT |

**Supplementary Table 2. List of the antibodies used for flow cytometry.**

| Antibody | Clone | Cat. No. | Vendor | Titer |
| --- | --- | --- | --- | --- |
| Blood leukocytes | | | | |
| CD45-PE | 30-F11 | 103106 | Biolegend  (San Diego, USA) | 1:25 |
| Ly6G-BV650 | 1A8 | 127641 | Biolegend  (San Diego, USA) | 1:12.5 |
| CD11b-PE/Dazzle | M1/70 | 101256 | Biolegend  (San Diego, USA) | 1:100 |
| Ly-6C-APC-Cy7 | HK1.4 | 128026 | Biolegend  (San Diego, USA) | 1:200 |
| CD115-BV711 | AFS98 | 135515 | Biolegend  (San Diego, USA | 1:50 |
| Blood lymphocytes | | | | |
| CD19-PacBl | 6D5 | 115523 | Biolegend  (San Diego, USA) | 1:12.5 |
| CD3-AF700 | 17A2 | 100216 | Biolegend  (San Diego, USA) | 1:50 |
| CD8a-AF647 | 53-6.7 | 100724 | Biolegend  (San Diego, USA) | 1:100 |
| CD4-FITC | RM4-5 | 11-0042-82 | Biolegend  (San Diego, USA) | 1:200 |
| Aortic leukocytes | | | | |
| Live Dead aqua 526 |  | L34965 | Thermo Fisher Scientific,  Eugene, USA | 1:25 |
| CD45-AF 700 | 30-F11 | 103128 | Biolegend  (San Diego, USA) | 1:25 |
| Ly-6G-BV421 | 1A8 | 127627 | Biolegend  (San Diego, USA) | 1:25 |
| CD11b-PerCP-Cy5-5 | M1/70 | 101228 | Biolegend  (San Diego, USA) | 1:25 |
| F4/80-PE | BM8 | 123110 | Biolegend  (San Diego, USA) | 1:25 |
| CD64-APC | X54-5/7.1 | 139306 | Biolegend  (San Diego, USA) | 1:25 |

**Supplementary Table 3. Characterization of immune cell populations quantified by flow cytometry in circulating blood and aorta.**

| **Circulating immune cell populations** | **Markers** |
| --- | --- |
| **Myeloid Leukocytes** | CD45+CD11b+ |
| **Lymphocytes** | CD45^+^CD19^+^CD3^+^ |
| **Monocytes** | CD45^+^CD11b^+^Ly6G^-^CD115^+^ |
| **Ly6C^high^ Monocytes** | CD45^+^CD11b ^+^CD115^+^Ly6C^high^ |
| **Ly6C^low^ Monocytes** | CD45^+^CD11b ^+^CD115^+^Ly6C^low^ |
| **Neutrophils** | CD45^+^CD11b^+^CD115^-^Ly6G^+^ |
| **CD4^+^ T cells** | CD45^+^CD19^+^ Ly6G^-^CD11b^-^CD19^-^CD3^+^CD4^+^ |
| **CD8a^+^ T cells** | CD45^+^CD19^+^Ly6G^-^CD11b^-^CD19^-^CD3^+^CD4^-^CD8a^+^ |
| **B cells** | CD45^+^CD19^+^ Ly6G^-^CD11b^-^CD19^+^ |
| **Aortic immune cell populations** | Markers |
| **Myeloid Leukocytes** | CD45+CD11b+ |
| **Lymphocytes** | CD45^+^Ly6G^-^CD11b^-^ |
| **Neutrophils** | CD45^+^CD11b^+^Ly6G^+^ |
| **Macrophages** | CD45^+^CD11b^+^Ly6G^-^F4/80^+^CD64^+^ |

**Supplementary Table 4. Canonical Pathway Agranulocyte adhesion and diapedesis Genelist.**

| **Symbol** | **Entrez Gene Name** | **Gene Symbol - mouse (Entrez Gene)/Ensembl** | **Expr Fold Change** | **Expr p-value** |
| --- | --- | --- | --- | --- |
| **Cxcl3** | C-X-C motif chemokine ligand 3 | Cxcl3 | 27,163 | 3,06E-07 |
| **ACTA1** | actin alpha 1, skeletal muscle | Acta1 | 5,217 | 1,46E-05 |
| **MYH4** | myosin heavy chain 4 | Myh4 | 18,866 | 2,64E-05 |
| **MYH2** | myosin heavy chain 2 | Myh2 | 6,895 | 2,06E-04 |
| **Cxcl2** | C-X-C motif chemokine ligand 2 | Cxcl2 | 7,385 | 4,71E-04 |
| **CXCR2** | C-X-C motif chemokine receptor 2 | Cxcr2 | 5,522 | 6,77E-04 |
| **MYH1** | myosin heavy chain 1 | Myh1 | 4,902 | 8,13E-04 |
| **IL36G** | interleukin 36 gamma | Il36g | 6,385 | 1,45E-03 |
| **Ppbp** | pro-platelet basic protein | Ppbp | 4,962 | 2,20E-03 |
| **MMP9** | matrix metallopeptidase 9 | Mmp9 | 3,332 | 2,36E-03 |
| **MYL1** | myosin light chain 1 | Myl1 | 1,654 | 2,41E-03 |
| **MYH6** | myosin heavy chain 6 | Myh6 | -3,138 | 3,79E-03 |
| **MMP12** | matrix metallopeptidase 12 | Mmp12 | 2,033 | 8,01E-03 |
| **CCL24** | C-C motif chemokine ligand 24 | Ccl24 | 2,423 | 9,18E-03 |
| **Cxcl5** | C-X-C motif chemokine ligand 5 | Cxcl5 | 3,607 | 1,00E-02 |
| **Ccl4** | C-C motif chemokine ligand 4 | Ccl4 | 2,954 | 1,80E-02 |
| **Ccl12** | C-C motif chemokine ligand 12 | Ccl12 | 1,722 | 3,06E-02 |
| **SELL** | selectin L | Sell | 1,912 | 3,30E-02 |
| **IL1RN** | interleukin 1 receptor antagonist | Il1rn | 1,995 | 3,34E-02 |
| **MYH7** | myosin heavy chain 7 | Myh7 | 2,863 | 4,94E-02 |

**Supplementary Table 5. Canonical Pathway Granulocyte Adhesion and Diapedesis Genelist.**

| **Symbol** | **Entrez Gene Name** | **Gene Symbol - mouse (Entrez Gene)/Ensembl** | **Expr Fold Change** | **Expr p-value** |
| --- | --- | --- | --- | --- |
| **Cxcl3** | C-X-C motif chemokine ligand 3 | Cxcl3 | 27,163 | 3,06E-07 |
| **Cxcl2** | C-X-C motif chemokine ligand 2 | Cxcl2 | 7,385 | 4,71E-04 |
| **CXCR2** | C-X-C motif chemokine receptor 2 | Cxcr2 | 5,522 | 6,77E-04 |
| **IL36G** | interleukin 36 gamma | Il36g | 6,385 | 1,45E-03 |
| **Ppbp** | pro-platelet basic protein | Ppbp | 4,962 | 2,20E-03 |
| **MMP9** | matrix metallopeptidase 9 | Mmp9 | 3,332 | 2,36E-03 |
| **MMP12** | matrix metallopeptidase 12 | Mmp12 | 2,033 | 8,01E-03 |
| **CCL24** | C-C motif chemokine ligand 24 | Ccl24 | 2,423 | 9,18E-03 |
| **Cxcl5** | C-X-C motif chemokine ligand 5 | Cxcl5 | 3,607 | 1,00E-02 |
| **Ccl4** | C-C motif chemokine ligand 4 | Ccl4 | 2,954 | 1,80E-02 |
| **Ccl12** | C-C motif chemokine ligand 12 | Ccl12 | 1,722 | 3,06E-02 |
| **SELL** | selectin L | Sell | 1,912 | 3,30E-02 |
| **IL1RN** | interleukin 1 receptor antagonist | Il1rn | 1,995 | 3,34E-02 |
| **IL1R2** | interleukin 1 receptor type 2 | Il1r2 | 2,552 | 3,64E-02 |
| **CSF3R** | colony stimulating factor 3 receptor | Csf3r | 2,056 | 4,27E-02 |

**Supplementary Table 6. Endothelial gene expression of 53 differentially expressed genes in the aorta.**

|  |  | **log2Fc** | **adj.p** |
| --- | --- | --- | --- |
| **Adrb3** | ENSMUSG00000031489 | -0,003 | 0,999 |
| **Sp100** | ENSMUSG00000026222 | 0,201 | 0,999 |
| **H2-T23** | ENSMUSG00000067212 | 0,057 | 0,999 |
| **Cebpb** | ENSMUSG00000056501 | -0,050 | 0,999 |
| **Ly6e** | ENSMUSG00000022587 | -0,019 | 0,999 |
| **Spon1** | ENSMUSG00000038156 | 0,164 | 0,999 |
| **Gbp3** | ENSMUSG00000028268 | -0,056 | 0,999 |
| **Irgm2** | ENSMUSG00000069874 | -0,608 | 0,999 |
| **Sncg** | ENSMUSG00000023064 | 0,600 | 0,999 |
| **Bst2** | ENSMUSG00000046718 | -0,048 | 0,999 |
| **Lypd8l** | ENSMUSG00000037145 | -0,025 | 0,999 |
| **Xaf1** | ENSMUSG00000040483 | -0,141 | 0,999 |
| **Mid1** | ENSMUSG00000035299 | -0,076 | 0,999 |
| **Trim34a** | ENSMUSG00000056144 | 0,086 | 0,999 |
| **Nnt** | ENSMUSG00000025453 | 0,114 | 0,999 |
| **Klf15** | ENSMUSG00000030087 | -0,532 | 0,999 |
| **Lgals3bp** | ENSMUSG00000033880 | -0,234 | 0,999 |
| **Apod** | ENSMUSG00000022548 | 0,133 | 0,999 |
| **Oas2** | ENSMUSG00000032690 | -0,165 | 0,999 |
| **Isg15** | ENSMUSG00000035692 | 0,058 | 0,999 |
| **Ifit3** | ENSMUSG00000074896 | -0,116 | 0,999 |
| **Oas1a** | ENSMUSG00000052776 | -0,425 | 0,999 |
| **Slfn8** | ENSMUSG00000035208 | 0,007 | 0,999 |
| **Oasl2** | ENSMUSG00000029561 | -0,362 | 0,999 |
| **Hsd11b1** | ENSMUSG00000016194 | -0,082 | 0,999 |
| **Zbp1** | ENSMUSG00000027514 | -0,399 | 0,999 |
| **Ifit1** | ENSMUSG00000034459 | -0,697 | 0,999 |
| **Usp18** | ENSMUSG00000030107 | 0,007 | 0,999 |
| **Rtp4** | ENSMUSG00000033355 | -0,419 | 0,999 |
| **Xlr3b** | ENSMUSG00000073125 | 0,207 | 0,999 |
| **Irf7** | ENSMUSG00000025498 | -0,325 | 0,999 |
| **Lep** | ENSMUSG00000059201 | 0,473 | 0,999 |
| **Oas3** | ENSMUSG00000032661 | -0,150 | 0,999 |
| **Oasl1** | ENSMUSG00000041827 | -0,329 | 0,999 |
| **Ifi206** | ENSMUSG00000037849 | -0,024 | 0,999 |
| **Slfn1** | ENSMUSG00000078763 | -0,038 | 0,999 |
| **Fam217a** | ENSMUSG00000021414 | -0,112 | 0,999 |
| **Acta1** | ENSMUSG00000031972 | -0,500 | 0,999 |
| **Tnni2** | ENSMUSG00000031097 | -0,313 | 0,999 |
| **Tnnc2** | ENSMUSG00000017300 | -0,091 | 0,999 |
| **Slfn4** | ENSMUSG00000000204 | 0,121 | 0,999 |
| **Atp2a1** | ENSMUSG00000030730 | 0,113 | 0,999 |
| **Myh4** | ENSMUSG00000057003 | 0,315 | 0,999 |
| **Cxcl3** | ENSMUSG00000029379 | 0,003 | 0,999 |
| **Gm4724** | ENSMUSG00000078897 | 0,005 | 0,999 |
| **Ifi208** | ENSMUSG00000066677 | -0,154 | 0,999 |
| **Chst4** | ENSMUSG00000035930 | -0,906 | 0,957 |
| **Fosb** | ENSMUSG00000003545 | 0,286 | 0,999 |
| **Arsk** | ENSMUSG00000021592 | 0,250 | 0,999 |
| **Flnc** | ENSMUSG00000068699 | -0,706 | 0,999 |
| **Zbed6** | ENSMUSG00000094410 | -0,210 | 0,999 |
| **Sgms2** | ENSMUSG00000050931 | 0,128 | 0,999 |
| **Atp1b1** | ENSMUSG00000026576 | 0,035 | 0,999 |
